# Methylammonium Tetrel Halide Perovskite Ion Pairs and Their Dimers: The Interplay between the Hydrogen-, Pnictogen- and Tetrel-Bonding Interactions

**DOI:** 10.3390/ijms241310554

**Published:** 2023-06-23

**Authors:** Pradeep R. Varadwaj, Arpita Varadwaj, Helder M. Marques, Koichi Yamashita

**Affiliations:** 1 Department of Chemical System Engineering, School of Engineering, The University of Tokyo, 7-3-1, Tokyo 113-8656, Japan; 2 School of Chemistry, Molecular Sciences Institute, University of the Witwatersrand, Johannesburg 2050, South Africa

**Keywords:** methylammonium tetrel halide perovskites, ion pair chemistry, resemblance between the gas and crystalline systems, charge-assisted hydrogen bonds, pnictogen bond, tetrel bond, stability and energetics, MESP, IGM-*δg^inter^*, NBO and QTAIM analyses

## Abstract

The structural stability of the extensively studied organic–inorganic hybrid methylammonium tetrel halide perovskite semiconductors, MATtX_3_ (MA = CH_3_NH_3_^+^; Tt = Ge, Sn, Pb; X = Cl, Br, I), arises as a result of non-covalent interactions between an organic cation (CH_3_NH_3_^+^) and an inorganic anion (TtX_3_^−^). However, the basic understanding of the underlying chemical bonding interactions in these systems that link the ionic moieties together in complex configurations is still limited. In this study, ion pair models constituting the organic and inorganic ions were regarded as the repeating units of periodic crystal systems and density functional theory simulations were performed to elucidate the nature of the non-covalent interactions between them. It is demonstrated that not only the charge-assisted N–H···X and C–H···X hydrogen bonds but also the C–N···X pnictogen bonds interact to stabilize the ion pairs and to define their geometries in the gas phase. Similar interactions are also responsible for the formation of crystalline MATtX_3_ in the low-temperature phase, some of which have been delineated in previous studies. In contrast, the Tt···X tetrel bonding interactions, which are hidden as coordinate bonds in the crystals, play a vital role in holding the inorganic anionic moieties (TtX_3_^−^) together. We have demonstrated that each Tt in each [CH_3_NH_3_^+^•TtX_3_^−^] ion pair has the capacity to donate three tetrel (σ-hole) bonds to the halides of three nearest neighbor TtX_3_^−^ units, thus causing the emergence of an infinite array of 3D TtX_6_^4−^ octahedra in the crystalline phase. The TtX_4_^4−^ octahedra are corner-shared to form cage-like inorganic frameworks that host the organic cation, leading to the formation of functional tetrel halide perovskite materials that have outstanding optoelectronic properties in the solid state. We harnessed the results using the quantum theory of atoms in molecules, natural bond orbital, molecular electrostatic surface potential and independent gradient models to validate these conclusions.

## 1. Introduction

Metal halide perovskite semiconductors are an important class of hybrid chemical systems [1,2,3]. They have been synthesized as either organic–inorganic hybrid perovskites [4,5,6] or all-inorganic perovskites [7,8,9]. Compounds of the first type have often been referred to as organometallic halide perovskites [10,11,12]. Since this is potentially misleading, Angelis and Kamat [13] have suggested referring to them as metal halide perovskites, following the arguments developed by one of us [14], although the practice of referring to them as organometallic halide perovskites continues [15,16,17,18,19].

In organic–inorganic hybrid perovskites [4,5,6], the organic cation couples with the inorganic anion to form an ion pair [20,21,22,23,24]; applying periodic boundary conditions leads to the formation of a bulk material [25,26]. In the case of all-inorganic perovskites, an inorganic cation couples with another inorganic anion to form an ion pair [9,20]. Both types of metal-based halide perovskites have been synthesized as crystalline materials in a variety of dimensions (i.e., 0D, 1D, 2D and 3D) [27,28,29,30]. Many of them have been characterized as 3D single metal halide perovskites [31,32,33]. Double metal halide perovskites [34,35,36] are predominantly all-inorganic hybrids [37,38,39,40,41].

Single metal halide perovskites are represented by the generic formula ABX_3_ [33,42] (or AMX_3_ [43,44,45]), where A is an organic or inorganic cation, B or M is a metal cation, and X is a halogen derivative. The oxidation states of A, B (or M) and X are +1, +2 and –1, respectively. Some popular organic–inorganic hybrids that have applications in optoelectronics are MATtX_3_ and FATtX_3_ (MA = CH_3_NH_3_^+^, FA = HC(NH_2_)_2_^+^ [46,47]; Tt = Pb [48,49,50,51], Sn [49,52,53]; X = I, Br). All-inorganic perovskites that have impressive optoelectronic properties include CsTtX_3_ [54,55] and RbTtX_3_ [56,57,58]. MAPbI_3_ and MAPbBr_3_ [59,60,61] have been applied in third-generation photovoltaics (efficient for photo-energy conversion).

The physical chemistry of many single metal halide perovskites has been investigated both experimentally and theoretically. Many views, not necessarily divergent, have been expressed about the optical and bandgap features of these systems [14,20,22,23,24,25,62,63,64]. Similar views have been provided on the interionic intermolecular interactions that act as the glue that holds the organic and inorganic materials together in organic–inorganic hybrid materials. The importance of organic cations as additives in organic–inorganic halide perovskites has been demonstrated [65,66], but there seems to be some controversy concerning the role they play in triggering their optoelectronic properties [9,19,21,67,68,69].

A point of much discussion is the nature of the chemical bonding in organic–inorganic hybrids, for example, in FAPbI_3_ [70] and MATtX_3_ (Tt = Pb; X = I, Br) [9,22,23,71,72,73]. Contrary to views that there is no hydrogen bonding in hybrid lead halide perovskites at room temperature [74], which is curious given that these systems have no geometric stability without the intermolecular interactions, we and others have stressed that the N–H···X hydrogen bonds hold the ion pairs MA CH_3_NH_3_^+^) and TtX_3_^−^ together in a well-defined structure of organic–inorganic hybrids, both in the low-temperature (orthorhombic [22,75,76]) and the room-temperature (tetragonal [23,77]) phases. The N–H···X hydrogen bonds formed by the methyl group of MA, as well as the C–N···X pnictogen bonds formed by the N end of the same cation with the halogen derivative of the inorganic moiety and their importance in the functionalization of these highly-valued perovskite systems, were either overlooked or ignored [22,23]. They have yet to be fully delineated both theoretically and experimentally.

**Figure 1:**
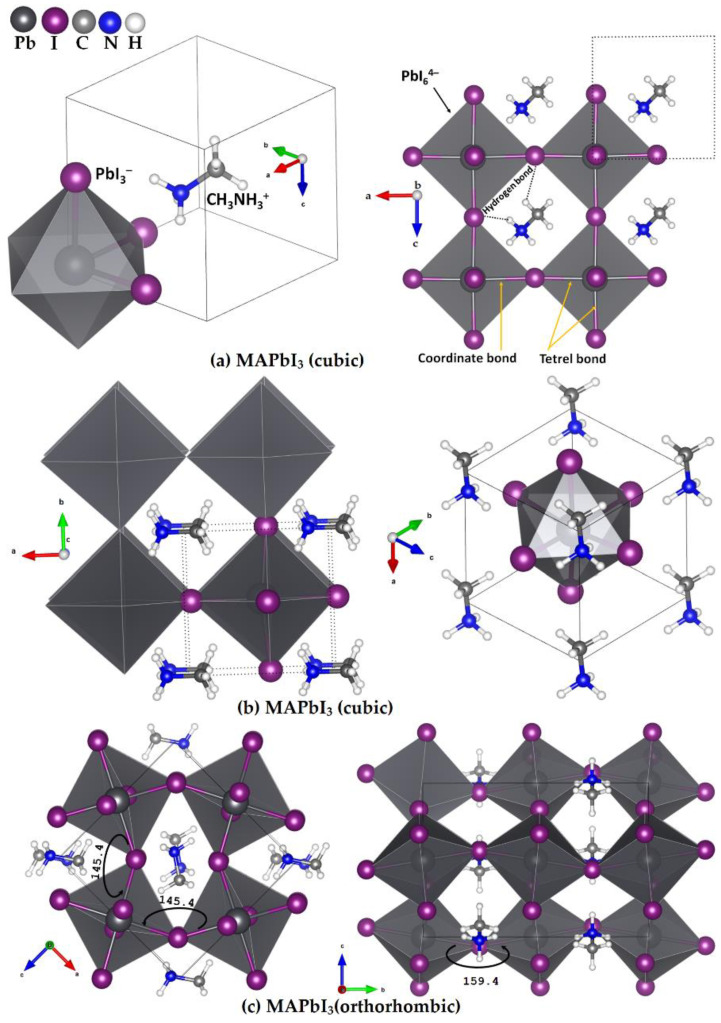
The unit cell (left) and 2 × 2 supercell (right) structure of cubic MAPbI_3_ in the high-temperature phase (*T* > 330 K [95]), showing the orientation of the organic cation along the (**a**) (111) and (**b**) (011) directions [25]. (**c**) Two different polyhedral views of the low-temperature orthorhombic phase of fully relaxed (PBEsol) geometry of MAPbI_3,_ with the titling angle (in degrees), ∠Pb–I–Pb, along two crystallographic directions (see ESI for details), obtained using the VASP code (version 5.4) [96,97,98,99,100]. Each polyhedron represents the PbI_6_^4−^ octahedron. Three types of bonds (coordinate, tetrel and hydrogen bonds) are marked in the polyhedral model in (**a**).

The aim of this study is to elucidate the types of intermolecular interactions between CH_3_NH_3_^+^ and TtX_3_^−^, which are the building blocks of CH_3_NH_3_TtX_3_ perovskites in the crystalline phase. In particular, we show using the [CH_3_NH_3_^+^•TtX_3_^−^] ion pair models that the charge-assisted N–H···X and C–H···X hydrogen bonds are important (geometric) synthons responsible for stabilizing the geometry of the inorganic [TtX_3_^−^] framework in the various phases of the system observed in the solid state (viz. the cubic phase of CH_3_NH_3_PbI_3_, Figure 1a,b). The C–N···X pnictogen bonds are non-negligible contributors to the tilting of the TtX_6_^4−^ octahedra of the low-temperature orthorhombic phase (Figure 1c) and the X–Tt···X tetrel bonds are the key geometric players in the formation of the TtX_6_^4−^ octahedra and the resultant cage-like structure that hosts the organic cation (see polyhedral models in Figure 1a–c). The definition and characteristic features of the hydrogen bond [78], pnictogen bond [79,80] and tetrel bond [9,81,82] have been discussed elsewhere, which we outline in a following section, and a discussion of the physical chemistry of charge-assisted non-covalent interactions can be found elsewhere [82,83,84,85,86]. We utilize Density Functional Theory (DFT) calculations at the *ω*B97X-D [87] level of theory, together with the Quantum Theory of Atoms in Molecules (QTAIM) [88], Molecular Electrostatic Surface Potential (MESP) [89,90,91,92] and Independent Gradient Model (IGM) [93,94] approaches, to validate our conclusions.

## 2. Results

### 2.1. Methylammonium Tetrel Halide Perovskite Ion Pairs

The (111) direction of the organic cation in the crystalline cubic structure of MAPbI_3_ results when the ammonium end of the cation faces a triangular face (formed by three iodides) of a [TtI_6_^4−^] octahedron of an extended solid (Figure 1a). When the unit cell shown in Figure 1a (left) is expanded, the methyl end of the diagonally placed MA faces the triiodide face of a [TtI_6_^4−^] that occupies the opposite corner of the cube (Figure 1a (right)); this also represents the (111) direction of the organic cation. The resulting structure when the organic cation is oriented along the (011) direction is shown in Figure 1b (right). A very similar orientation of the organic cation is likely to be obtained when X = Br (or Cl).

The orientation of the organic cation in cubic MAPbI_3_ plays a fundamental role in determining the material optoelectronic properties of methylammonium tetrel halide perovskites [25]. For instance, when MA orientates along the (011) direction (Figure 1b), the inorganic octahedral cage distorts and the bandgap of cubic MAPbI_3_ becomes indirect; when the organic cation is oriented along the (111) direction, there is no distortion of the inorganic octahedral cage with the PbI_6_^4−^ units linearly bonded to each other through the octahedral edges, and the material has a direct bandgap. Using the results of periodic DFT calculations [25], it was shown that the relaxation of the halide perovskite lattice with the molecular cation as shown in Figure 1a (left) does not distort its orientation in any crystallographic direction. This was not the case when MA initially orientated in the (001) direction; the relaxation of the lattice produced a structure with MA orientated along the (011) or an equivalent direction. Both configurations were local minima, with an energy difference of 20 meV in favor of the lattice that has the organic cation along the (011) direction [25].

**Figure 2:**
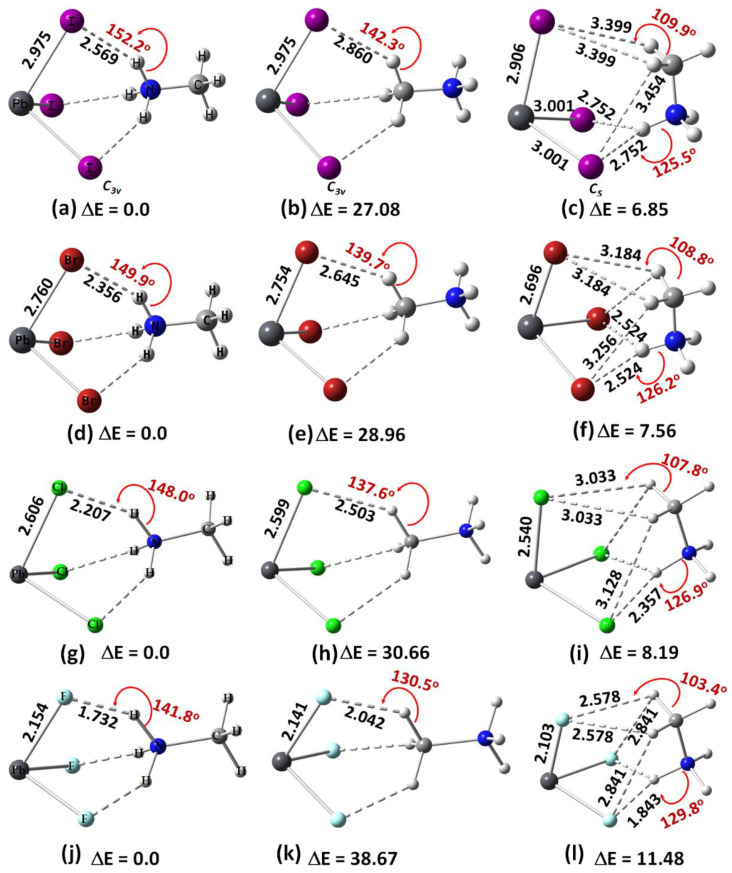
[*ω*B97X-D/def2-TZVPPD] level fully relaxed geometries of [CH_3_NH_3_^+^•PbX_3_^−^] (X = F, Cl, Br, I) ion pairs, including (**a**) [I_3_Pb···NH_3_CH_3_]; (**b**) [I_3_Pb···CH_3_NH_3_]; (**c**) [I_3_Pb···H_3_NCH_3_]; (**d**) [Br_3_Pb···NH_3_CH_3_]; (**e**) [Br_3_Pb···CH_3_NH_3_]; (**f**) [Br_3_Pb···H_3_NCH_3_]; (**g**) [Cl_3_Pb···NH_3_CH_3_]; (**h**) [Cl_3_Pb···CH_3_NH_3_]; (**i**) [Cl_3_Pb···H_3_NCH_3_]; (**j**) [F_3_Pb···NH_3_CH_3_]; (**k**) [F_3_Pb···CH_3_NH_3_]; (**l**) [F_3_Pb···H_3_NCH_3_]. Shown are selected coordinate and hydrogen bond distances (solid and dotted lines, respectively) in Å and hydrogen bond angles in degrees. The relative energies (Δ*E*) with respect to the most stable conformer for each series with a given halogen derivative are shown in kcal mol^−1^. Atom labeling is shown for selected systems.

A question that arises is whether the orientations of MA observed in the high-temperature phase of the system also occur in the gas phase when there are no boundary conditions. To address this question, we examined the geometries of [CH_3_NH_3_^+^•PbI_3_^−^] ion pairs in the gas phase (Figure 2a–c). The first two panels, Figure 2a,b, show that the ammonium and methyl ends of MA face the triangular iodide face of the anion, respectively, and they mimic the (111) orientation of the cation in the periodic structure (Figure 1a). The third configuration of [CH_3_NH_3_^+^•PbI_3_^−^] (Figure 2c) resembles the (011) orientation of MA in the solid state (Figure 1b). The corresponding conformations were also identified for [CH_3_NH_3_^+^•PbBr_3_^−^] (Figure 2d–f), [CH_3_NH_3_^+^•PbCl_3_^−^] (Figure 2g–i) and [CH_3_NH_3_^+^•PbF_3_^−^] (Figure 2j–l). We will refer to the first two conformations of each ion pair type on the left of each panel, which mimic the (111) direction of the organic cation, as Conf. 1 and 2, respectively; the conformation on the right of each panel, which mimics the (011) orientation of the cation, will be referred to as Conf. 3. In each case, it was found that Conf. 1 is more stable than Conf. 2 or Conf. 3 (relative values of Δ*E* are shown in Figure 2), i.e., Conf. 1 > Conf. 3 > Conf. 2. Conf. 1 is calculated to be the only minimum, while Conf. 2 and 3 are, respectively, second- and first-order transition state gas phase structures.

The orientation of the organic cation inside the inorganic cage (Figure 1a, right) determines the nature of the charge-assisted hydrogen bonding between it and the cage. When it lies along the (111) orientation, the three H atoms of the ammonium end (or the methyl end) of MA are involved in three equivalent N–H···I (or C–H···I) hydrogen bonds with the three I atoms of the I_3_ of [PbI_3_^−^]; see for example, Figure 2a,b, for [I_3_Pb···NH_3_CH_3_] (Conf. 1) and [I_3_Pb···CH_3_NH_3_] (Conf. 2), respectively. When the cation is oriented along the (011) direction, the N–H···I/C–H···I hydrogen bonds of MA with the [PbI_3_^−^] face are not equivalent; the two C–H···I (equivalent) hydrogen bonds are longer than the N–H···I hydrogen bonds (see, for example, Conf. 3 ([I_3_Pb···H_3_NCH_3_]), Figure 2c). The same trend was found when X = Br, Cl and F (see Figure 2d–f, g–i and j–l, respectively).

For a given halogen derivative in [CH_3_NH_3_^+^•PbX_3_^−^], the N–H···X hydrogen bond distances are shorter than the C–H···X hydrogen bonds, in agreement with what was observed in the solid state for CH_3_NH_3_PbX_3_ (X = Cl, Br, I) perovskites [22,23]. This means that the charge-assisted N–H···X hydrogen bonds formed by the ammonium end of MA are stronger than the C–H···X hydrogen bonds formed by the methyl end of MA, and their strength increases with the electronegativity of the halogen (F > Cl > Br > I). For both Conf. 1 and Conf. 2, the hydrogen bond distance decreases as the halogen becomes more electron-withdrawing (see Figure 2a,d,g,j or Figure 2b,e,h,k), a feature found for ion pairs when Tt = Sn and Ge, but not when Tt = Si (see Figures S1–S4 of the Electronic Supplementary Information, ESI). In all cases, the hydrogen bonds are non-linear (∠N–H···X or ∠C–H···X < 143°), regardless of their type, and (based on the bond lengths) the strength of the Pb–X coordinate bonds decreases passing from F through Br to Cl to I in [CH_3_NH_3_^+^•PbX_3_^−^] (see Figure 2a,d,g,h, for example).

Replacement of Pb by Sn and Ge in [CH_3_NH_3_^+^•PbX_3_^−^] did not appreciably change the nature of the coordination between Tt and X in TtX_3_^−^, although the hydrogen-bonded environment was affected (see Figures S1a–l and S2a–l of the ESI). When Tt = Si in [CH_3_NH_3_^+^•TtX_3_^−^], Conf. 3 was found to be unstable for X = I and Br (see Figure S3a–d), but [X_3_Si···NH_3_CH_3_] was more stable than [X_3_Si···CH_3_NH_3_]. Figures S3e–g and S4a-c of the ESI provide the geometric and relative energy details of the ion pair series [CH_3_NH_3_^+^•SiX_3_^–^] and [CH_3_NH_3_^+^•SiF_3_^–^], respectively. In all cases, Conf. 1 was observed to be more stable than Conf. 2 and Conf. 3.

Conf. 3 was obtained from the initial geometry shown in Figure S4d for each of the three tetrel halide perovskite ion pair series, [CH_3_NH_3_^+^•PbX_3_^−^] (X = Cl, Br, I). However, when this initial configuration was used for relaxation of the [CH_3_NH_3_^+^•SiF_3_^−^] ion pair, the resulting geometry was no longer an ion pair and the initial configuration was not retained after energy minimization. This was not the case when the methyl and ammonium ends of the organic cation were pointed toward the center of the trifluoride triangular face of SiF_3_^−^. The energy-minimized geometry in Figure S4d shows that there is a significant interaction between the two ionic moieties when in close proximity, with a breaking of the N–H covalent bond and the formation of two neutral species, SiHF_3_ and CH_3_NH_2_, due to complete hydrogen transfer from the ammonium end to the anion. (This is addressed later.) Due to this, we then used the optimized geometry of the [PbF_3_^−^···NH_3_CH_3_^+^] ion pair (Figure 2l) as an initial configuration, replacing Pb with Si; the final energy-minimized geometry is shown in Figure S4c. Although this was the case for the system with the anion SiF_3_^−^, a similar attempt did not stabilize the analogous geometry of the ion pair when X = I and Br.

To help determine the validity of these conclusions we performed an MESP analysis; the results are summarized in Table 1. Analysis of these data suggests that the Si surfaces of [SiX_3_^−^···NH_3_CH_3_^+^] (X = I and Br) ion pairs are weakly positive but are negative in the remaining eight ion pairs of the same series regardless of the identity of the halogen derivative. The halogen in [SiX_3_^−^] has high electron density compared to the surface of the Si atom. There are three equivalent negative σ-holes on Si in each of the two ion pairs for a given halogen except for Conf. 3, and their strength decreases with an increase in the size of the halogen in [SiX_3_^−^]. The σ-hole on Si is more electrophilic when the ammonium head faces the triangular X_3_ face of [SiX_3_^−^] than when the methyl head faces that face. This is reasonable since the –NH_3_ group in MA is strongly electrophilic and hence relatively more electron-withdrawing than the methyl head. The first arrangement causes the development of a structure with relatively greater depletion of charge density on the surface of the Si atom along the outermost extensions of the three X–Si bonds. Since the lone pair of the Si lies along the outer extension of the *C_3v_* axis, the *V_S,min_* associated with it is increasingly more positive as the halogen becomes more electron-withdrawing, making the entire surface of the Si atom nucleophilic.

**Figure 3:**
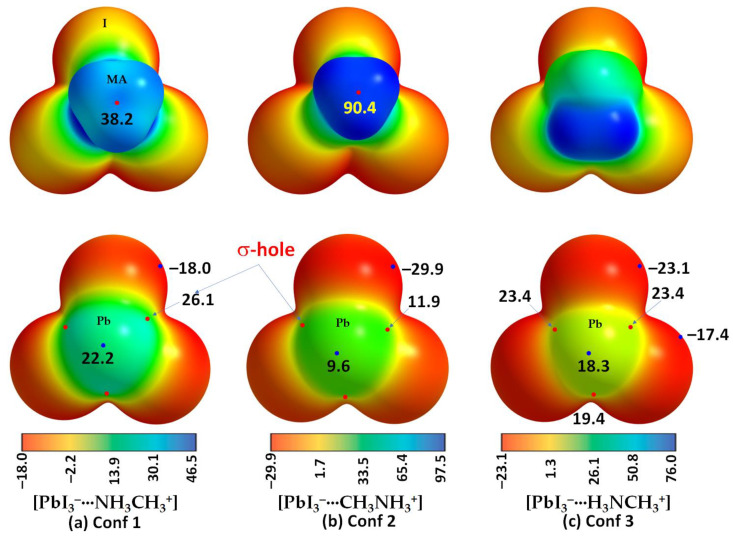
[*ω*B97X-D/def2-TZVPPD] level potential on the electrostatic surface of the three conformations of [CH_3_NH_3_^+^•PbI_3_^−^]: (**a**) [PbI_3_^−^···NH_3_CH_3_^+^]; (**b**) [PbI_3_^−^···CH_3_NH_3_^+^]; and (**c**) [PbI_3_^−^···H_3_NCH_3_^+^]. The methyl, ammonium and both groups of MA are facing the reader in the top panel of (**a**), (**b**) and (**c**), respectively. The Pb atom is facing the reader in the bottom panel of all three MESP plots shown in (**a**–**c**). Values on the color bar are in kcal mol^−1^. Atom labeling is shown for selected systems.

The electrostatic surfaces of Pb in [PbX_3_^−^···NH_3_CH_3_^+^], [PbX_3_^−^···CH_3_NH_3_^+^] and [PbX_3_^−^···H_3_NCH_3_^+^] have electron-deficient regions along the X–Pb bond extensions (*V_S,max_* (X–Pb) > 0) (Figure 3). They are equivalent in Conf. 1 or 2 but not in Conf. 3. The surface of Pb along the outermost extension of the *C_3v_* axis is also positive, represented by a positive local minimum of potential (*V_S,min_* (Pb) *>* 0). For example, values of *V_S,min_* (Pb) are 22.2, 9.6 and 18.3 kcal mol^−1^ for Conf. 1, Conf. 2 and Conf. 3, as shown in Figure 3a–c (bottom), respectively. These results indicate that the coordinately bonded Pb atom has three electrophilic σ-holes in these molecular ion pairs, and its surface is entirely electrophilic. An exception was found for [F_3_Pb^−^···CH_3_NH_3_^+^] when the 0.001 a.u. isoelectronic density envelope was invoked on which to compute the potential (*V_S,max_* (F–Pb) = −0.4 kcal mol^−1^), but a larger isoelectronic density envelope of 0.0015 a.u. gave *V_S,max_* (F–Pb) = 4.1 kcal mol^−1^. Both C and N atoms along the N–C and C–N bond extensions have electrophilic σ-holes in Conf. 1 and Conf. 2, respectively, and their strength in the former was weaker than that in the latter (*V_S,max_(*N–C) and *V_S,max_*(C–N) 38.2 and 90.4 kcal mol^−1^, respectively). Similarly, the lateral portions of the halogen in Conf. 1 or Conf. 2 are equipotential, described by *V_S,min_* (X) < 0, although this was not the case in Conf. 3 because of symmetry. For instance, the surface of the halogen that is hydrogen-bonded to the methyl group is less negative than the halogens bonded to the ammonium group in Conf. 3 (*V_S,min_* (X) −23.1 and −17.4 kcal mol^−1^, respectively). The detailed nature of the MESP graphs of the three conformations of [CH_3_NH_3_^+^•SnI_3_^−^] and [CH_3_NH_3_^+^•GeI_3_^−^] ion pairs are shown in Figures S5 and S6 of the ESI, respectively.

As observed in the case of methylammonium lead halide perovskite ion pairs, the tin surfaces of the three tin-based halide perovskite ion pairs (I_3_Sn···NH_3_CH_3_, I_3_Sn···H_3_NCH_3_ and Br_3_Sn···NH_3_CH_3_) are entirely positive, while the surfaces of the same atom in the remaining nine tin halide perovskite ion pairs are not completely positive; *V_S,min_* (Sn) is negative in the first two and negative in the latter nine ion pairs (Table 1). Except for F_3_Sn···CH_3_NH_3_, the three σ-holes on the surface of Sn along the X–Sn bond extensions are electrophilic (*V_S,max_*(X–Sn) > 0). They are negative for F_3_Sn···CH_3_NH_3_ regardless of the isoelectronic density envelopes used for mapping of the potential (cf. Table 1).

In the case of germanium halide perovskite ion pairs, *V_S,min_*(Ge) is found to be negative for all 12 ion pairs, evidence that the lone pair of Ge is active along the outer extension of the *C_3v_* axis. The σ-holes on the surface of Ge in I_3_Ge···CH_3_NH_3_ (X = I, Br, Cl) are not all nucleophilic, although they are for the germanium fluoride perovskite ion pairs (F_3_Ge···NH_3_CH_,_ F_3_Ge···CH_3_NH_3_ and F_3_Ge···H_3_NCH_3_). This suggests that the formation of an MAGeF_3_ perovskite is unlikely in the solid state, as inferred for the MASiX_3_ systems (vide supra). The synthesis of the chloro- and bromo-analogues of MAGeF_3_ may be feasible, although the interaction between the ion pairs will be weaker and thus it is expected that the solid-state compounds would be readily degraded.

Among the ion pairs investigated, the lead atom in Br_3_Pb···NH_3_CH_3_ provides the strongest σ-holes, suggesting that they are likely to form stronger oligomers, or clusters, when a number of such ion pairs are in close proximity. The phenomenon is likely to persist in the crystalline phase and rationalizes why MAPbBr_3_ perovskites were observed to be significantly more stable under ambient conditions than MAPbI_3_ perovskite materials [59,101,102]. A number of suggestions have been provided to explain the enhanced stability of MAPbBr_3_ compared to MAPbI_3_, including bromide migration, and the reduced activation energy, diffusion coefficient and concentration for halide ions in MAPbBr_3_ compared to MAPbI_3_ [59].

### 2.2. QTAIM, IGM-δg^inter^ and NBO Analysis of Ion Pairs

**Figure 4:**
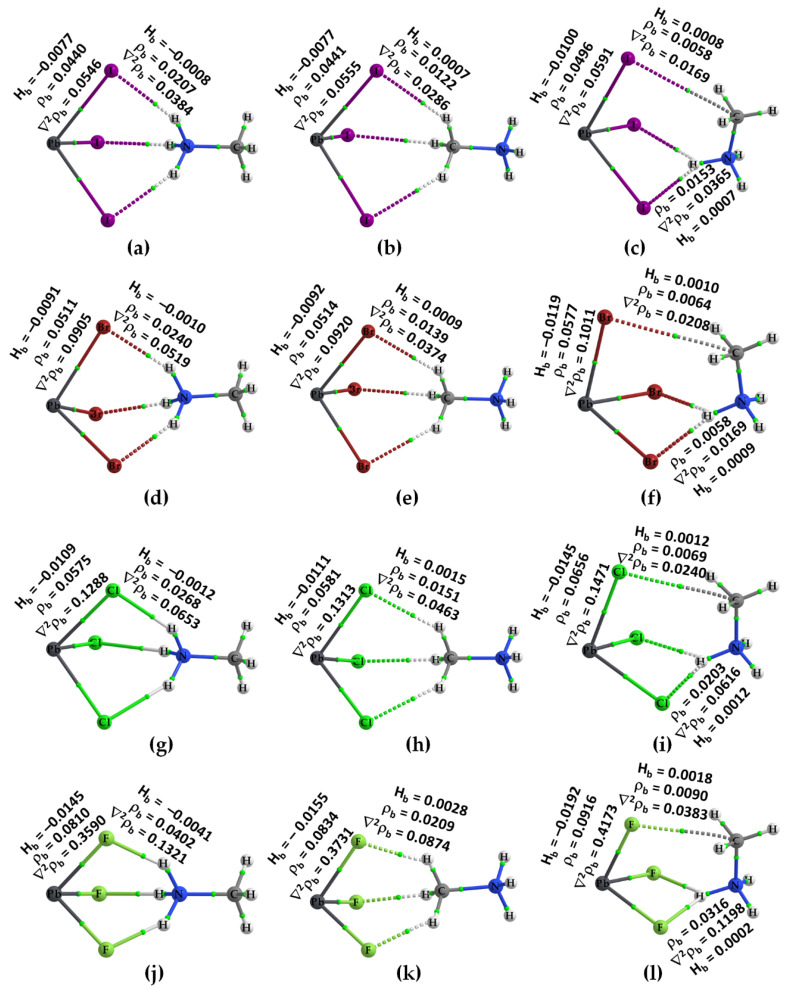
[*ω*B97X-D/def2-TZVPPD] level QTAIM’s molecular graphs for [CH_3_NH_3_^+^•PbX_3_^−^] (X = F, Cl, Br, I) ion pairs in the conformational space, including (**a**) [I_3_Pb···NH_3_CH_3_]; (**b**) [I_3_Pb···CH_3_NH_3_]; (**c**) [I_3_Pb···H_3_NCH_3_]; (**d**) [Br_3_Pb···NH_3_CH_3_]; (**e**) [Br_3_Pb···CH_3_NH_3_]; (**f**) [Br_3_Pb···H_3_NCH_3_]; (**g**) [Cl_3_Pb···NH_3_CH_3_]; (**h**) [Cl_3_Pb···CH_3_NH_3_]; (**i**) [Cl_3_Pb···H_3_NCH_3_]; (**j**) [F_3_Pb···NH_3_CH_3_]; (**k**) [F_3_Pb···CH_3_NH_3_]; (**l**) [F_3_Pb···H_3_NCH_3_]. The bond paths are shown as solid and dotted lines, respectively, in atom colors, and bond critical points (bcps) as tiny spheres in green between bonded atomic basins. Values (in a.u.) represent the charge density (*ρ*_b_), the Laplacian of the charge density (∇^2^*ρ*_b_) and the total energy density (*H*_b_) at bcps. Atom labeling is shown for each case.

The molecular graphs of all twelve ion pairs associated with the three conformers [PbX_3_^−^···NH_3_CH_3_^+^], [PbX_3_^−^···CH_3_NH_3_^+^] and [PbX_3_^−^···H_3_NCH_3_^+^] (X = I, Br, Cl, F) are shown in Figure 4. The N–H···X hydrogen bonds in Conf. 1 and Conf. 3 are shown in the ion pairs (Figure 4a,c,d,f,g,i,j,l) as dotted bond paths between the bonded atomic basins with a (3, –1) bond critical point (bcp) between them [78]. The C–H···X hydrogen bonds are present in Conf. 2 in which the methyl H atoms point towards the triangular X_3_ face of the inorganic anion. They are weaker than the N–H···X hydrogen bonds, indicated by the charge density values, *ρ*_b_, at their corresponding bcps.

QTAIM misses the bond path topologies of the C–H···X hydrogen bonds in the molecular graphs shown in Figure 4c,f,i,l; this is not unexpected since the intermolecular distances associated with these weakly bonded close contacts (cf. Figure 2c,f,i,l) are longer than the sum of the van der Waals radii of X and H (*r*_vdW_(H) = 1.20 Å; *r*_vdW_(F) = 1.46 Å; *r*_vdW_(Cl) = 1.82 Å; *r*_vdW_(Br) = 1.86 Å; *r*_vdW_(I) = 2.04 Å) [103]. Very similar results have been reported recently in a study that focused on the physical chemistry of anion–molecule systems driven by tetrel bonds [82]. QTAIM identifies a C···X tetrel bond between the methyl C of MA and an X site on PbX_3_^−^ in Conf. 3 (cf. Figure 4c,f,i,l), instead of C–H···X hydrogen bonds; the emergence of such an interaction is not surprising since the outer electrostatic surface of the C atom along the H–C bond extensions is positive.

The topological charge density characteristics of the intermolecular interactions identified by QTAIM are different from those of the Pb–X coordinate bonds. Most of the former, but not all, are characterized by ∇^2^*ρ*_b_ > 0 and *H*_b_ > 0, whereas the latter are characterized by ∇^2^*ρ*_b_ > 0 but *H*_b_ < 0 and a relatively large *ρ*_b_, where ∇^2^*ρ*_b_ and *H*_b_ are the Laplacian of the charge density and the total energy density, respectively. These results suggest that most of the intermolecular (interionic) interactions are of the closed-shell type [104], whereas the coordinate bonds in the ion pairs are of mixed character [105]. For each series with a given halogen derivative, the value of *ρ*_b_ is the largest for the N–H···X hydrogen bond (see Conf. 1 and Conf. 3) and the smallest for the C···X tetrel bond (Conf. 3). The largest value of *ρ*_b_ is associated with the N–H···X hydrogen bonds in some ion pairs, for which the bond paths between the interacting ions are described by solid lines in atom colors (viz. [Cl_3_Pb···NH_3_CH_3_] (Figure 4g) and F_3_Pb···NH_3_CH_3_] (Figure 4j)). The three equivalent N–H···X hydrogen bonds in Conf. 1 of each series possess some covalency character since *H*_b_ < 0 at the bcps [105] (see Figure 4a,d,g,j).

**Figure 5:**
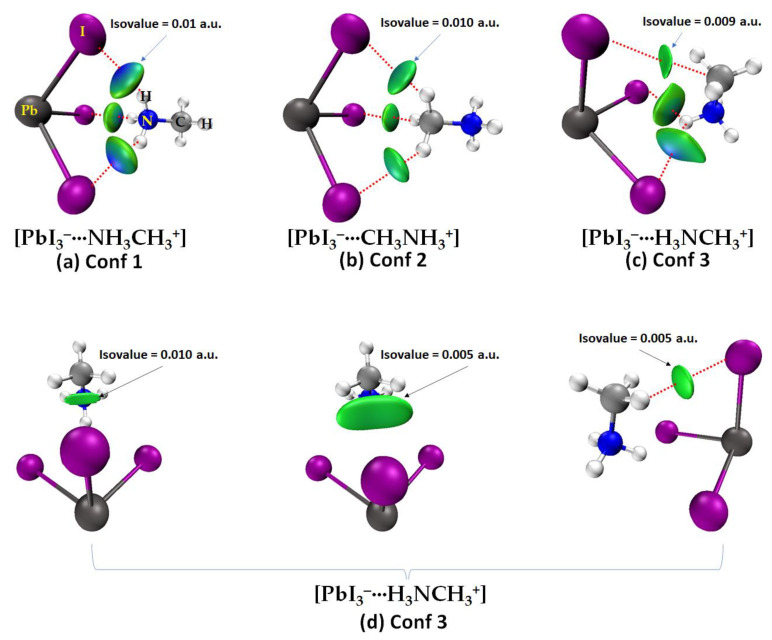
[*ω*B97X-D/def2-TZVPPD] level IGM-*δg^inter^* isosurface plots of the three conformations of [CH_3_NH_3_^+^•PbI_3_^−^]: (**a**) [PbI_3_^−^···NH_3_CH_3_^+^]; (**b**) [PbI_3_^−^···CH_3_NH_3_^+^]; (**c**) [PbI_3_^−^···H_3_NCH_3_^+^]. Shown in (**d**) are the IGM-*δg^inter^* isosurface plots for [PbI_3_^−^···H_3_NCH_3_^+^], in which different isovalues of IGM are shown. Labeling of selected atoms is shown in (**a**).

That QTAIM missed the C–H···X hydrogen bonds in Conf. 3 is confirmed by our IGM-*δg^inter^* analysis, presented in Figure 5. The results demonstrate that the IGM-*δg^inter^* isosurface volumes (circular or flat), colored bluish green (or green), spread within the area between the interacting atomic basins that are non-covalently engaged with each other, a color code that appears only when there is an attractive interaction between the interacting basins. These isosurfaces are representatives of *ρ* × sign (*λ*_2_) < 0, where *λ*_2_ is the second eigenvalue of the Hessian of the charge density matrix. When *ρ* × sign (*λ*_2_) > 0, one would expect repulsion between interacting basins, and these are generally described by red isosurfaces. Van der Waals interactions correspond to *ρ* × sign (*λ*_2_) ≈ 0 [93,94]. Clearly, as inferred from geometries alone (see Figure 2, for example), Conf. 1 and Conf. 2 are stabilized by N–H···I and C–H···I hydrogen bonds, respectively, with the former relatively stronger than the latter. Similarly, Conf. 3 is stabilized by N–H···I, C···I and C–H···I close contacts. The latter are very weak and appear only when an isovalue < 0.01 a.u. is used. The flat capsule type isosurface volume between the methyl group in MA and I in the inorganic anion in Figure 5d suggests that the attraction between the interacting units is weakly dispersive and includes weak C–H···I hydrogen bonds and the C···I tetrel bond. The presence of the former was confirmed when the interacting atomic basins (H and I) in the ion pair were used in the analysis of IGM-*δg^inter^* for which a smaller isovalue of 0.005 a.u. was invoked. This is shown in Figure 5d (right), and the IGM-*δg^inter^* isosurface volume between I and H representing C–H···I is more localized and weaker. These conclusions may hold for ion pairs formed with any other halogen derivative.

We carried out a second-order natural bond orbital analysis [106,107] to provide some insight into the nature of the charge transfer (hyperconjugative) interactions that occur between the “filled” (donor) Lewis-type NBOs and “empty” (acceptor) non-Lewis NBOs, but only for the three confirmations of [CH_3_NH_3_^+^•PbI_3_^−^] (Figure 5). The results suggest that the charge transfer interactions between the acceptor and donor NBOs responsible for either of the two N–H···I hydrogen bonds in Conf. 3 ([PbI_3_^−^···H_3_NCH_3_^+^]) can be described by *n*(3)I → σ*(N–H) (*E*^(2)^ = 6.71 kcal mol^−1^) and σ(Pb–I) → σ*(N–H) (*E*^(2)^ = 2.0 kcal mol^−1^), whereas the C–H···I hydrogen bond and H–C···I tetrel bond are described by *n*(3)I → σ*(N–C) (*E*^(2)^ = 0.17 kcal mol^−1^) and *n*(3)I → σ*(N–C) (*E*^(2)^ = 0.16 kcal mol^−1^), respectively, where *n*(3) refers the third lone pair and *E^(2)^* is the second-order stabilization energy [106,108]. The charge transfer interaction for each of the three N–H···I hydrogen bonds in Conf. 1 ([PbI_3_^−^···NH_3_CH_3_^+^]) is described by *n*(3)I → σ*(N–H) (*E*^(2)^ = 14.52 kcal mol^−1^), σ(Pb–I) → σ*(N–H) (*E*^(2)^ = 0.52 kcal mol^−1^) and *n*(3)I → σ*(N–C) (*E*^(2)^ = 0.36 kcal mol^−1^), with the latter indicative of a weak C–N···I pnictogen bond. Similarly, the charge transfer interaction associated with each of the three C–H···I hydrogen bonds in Conf. 2 [PbI_3_^−^···CH_3_NH_3_^+^] is described by *n*(3)I → σ*(C–H) (*E*^(2)^ = 4.17 kcal mol^−1^), and *n*(3)I → σ*(C–N) (*E*^(2)^ = 0.47 kcal mol^−1^), indicating that the σ* anti-bonding orbital of the C–N bond of the organic cation has the ability to act as an acceptor of electronic charge density via a charge transfer interaction with the π-type lone pair orbital of iodine in the inorganic anion and corresponds to an I–C···I tetrel bond. Since the geometric topology is very similar for all ion pairs investigated, it may be assumed that similar charge transfer interactions occur between the interacting ions that lead to the formation of the other ion pairs investigated.

### 2.3. Energy Stability of the Ion Pairs

The interaction energies Δ*E* of all the ion pairs investigated are summarized in Table 2; the total electronic energies obtained from the geometries of the ion pairs and the individual ions in the energy-minimized geometries of the same ion pairs were used. The counterpoise procedure of Boys and Bernardi [109] was followed, as implemented in Gaussian 16 [110] (see Section 4 for details). Among the three conformers examined for each tetrel derivative for a given halide, the hydrogen-bonded ion pair that has the ammonium head pointing towards the X_3_ triangular face of the anion was found to be the most stable. For example, the relative stability of the three conformers of [CH_3_NH_3_^+^•PbI_3_^−^] follows the order: Conf. 1 (I_3_Pb···NH_3_CH_3_) > Conf. 3 (I_3_Pb···H_3_NCH_3_) > Conf. 2 (I_3_Pb···CH_3_NH_3_) (Figure 2a–c), which is similar to that of their corresponding interaction energies, summarized in Table 1. The most stable ion pair was F_3_Pb···NH_3_CH_3_, with an interaction energy of −138.54 kcal mol^−1^ (Table 2).

The interaction energy listed in Table 2 for each ion pair is not the consequence of a single hydrogen bond. For example, this means that the Δ*E*(BSSE) of −104.85 kcal mol^−1^ for I_3_Pb···NH_3_CH_3_ arises from three equivalent charge-assisted I···H(N) hydrogen bonds; that of −76.33 kcal mol^−1^ for I_3_Pb···CH_3_NH_3_ is due to three equivalent charge-assisted I···H(C) hydrogen bonds; and that of −97.53 mol^−1^ for I_3_Pb···H_3_NCH_3_ is the result of a pair of equivalent charge-assisted I···H(N) hydrogen bonds and a pair of charge-assisted I···H(C) hydrogen bonds.

For ion pairs formed with a given tetrel derivative and a variable halide, i.e., [X_3_Pb···NH_3_CH_3_] where X = F, Cl, Br, I, the interaction energy increases (F > Cl > Br > I) as the polarizability of the halide decreases (I > Br > Cl > F). This is intuitively obvious since the electronegativity of the halides plays a significant role in determining the strength of an intermolecular charge-assisted hydrogen bonds.

While the interaction energies of the fluorinated ion pairs (viz. F_3_Pb···H_3_NCH_3_ and F_3_Sn···H_3_NCH_3_) are very large, and are of the ultra-strong type, these ion pairs hinder the formation of their corresponding perovskite structures in the solid state since the volume of the cage formed by the inorganic moiety is unlikely to be large enough to accommodate the organic cation.

On the other hand, the interaction between the two neutral molecules SiHF_3_ and CH_3_NH_2_ in F_3_HSi···NH_2_CH_3_ (Figure S4d) is not weak. The monomers in the complex are bonded to each other through a Si···N tetrel bond. The formation of this bond is perhaps obvious since the lone pair on the ammonium N is directly engaged with the positive σ-hole on the Si atom in SiHF_3_, which appears along the F–Si bond extension. The uncorrected (and BSSE-corrected) interaction energy of this bond is –22.80 (–22.23) kcal mol^−1^, indicative of a reasonably strong non-covalent interaction [81].

### 2.4. [CH_3_NH_3_^+^•TtX_3_^−^]_2_ (Tt = Si, Ge, Sn, Pb; X = F, Cl, Br, I) Dimers

**Figure 6:**
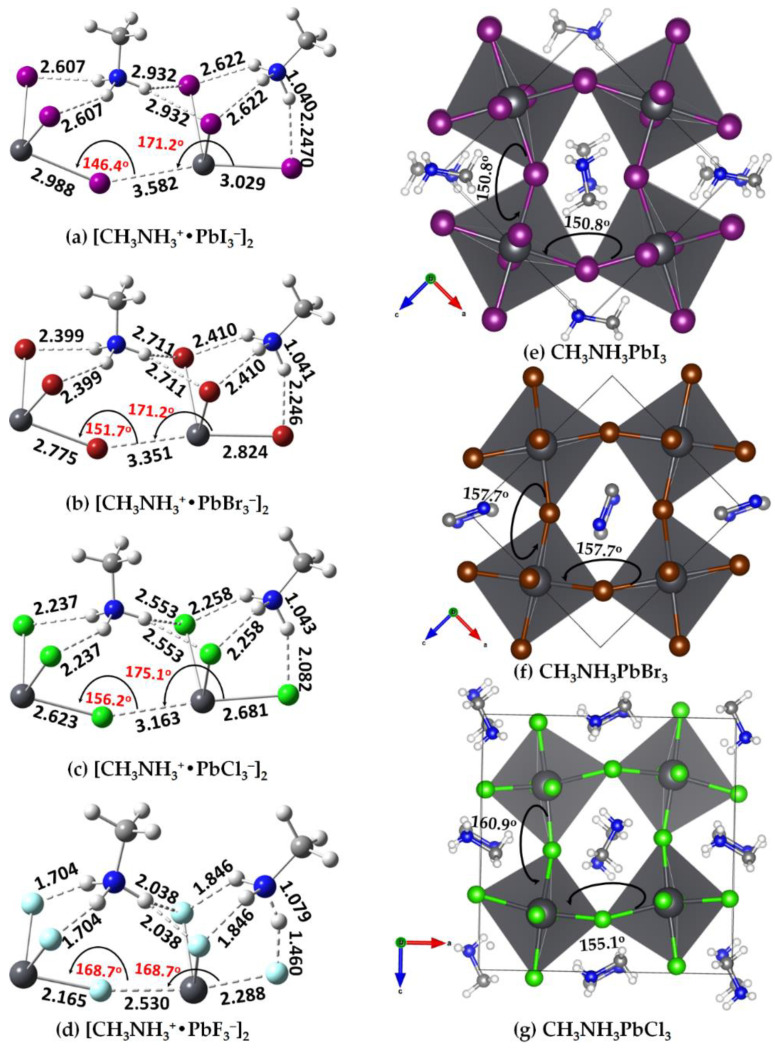
[*ω*B97X-D/def2-TZVPPD] level fully relaxed gas phase geometries of (**a**) [CH_3_NH_3_^+^•PbI_3_^−^]_2_; (**b**) [CH_3_NH_3_^+^•PbBr_3_^−^]_2_; (**c**) [CH_3_NH_3_^+^•PbCl_3_^−^]_2_; and (**d**) [CH_3_NH_3_^+^•PbF_3_^−^]_2_. Shown in (**d**–**f**) are the low-temperature orthorhombic structures of MAPbI_3_, MAPbBr_3_ and MAPbCl_3_. Selected bond lengths (Å) and bond angles (degrees) are shown in (**a**–**d**), and the tilting angle (degrees) is shown in (**e**–**g**). The H atoms in MA are missing in the crystal structure of MAPbBr_3_.

The fully relaxed geometries of [CH_3_NH_3_^+^•PbI_3_^−^]_2_ (X = F, Cl, Br,I) dimers are shown in Figure 6. The connectivity between the ion pairs is driven by three types of intermolecular interactions: the N–H···X hydrogen bonds, the C–N···X pnictogen bonds and the X–Pb···X tetrel bonds. A tetrel bond occurs when there is evidence of a net attractive interaction between an electrophilic region associated with a covalently or coordinately bonded tetrel atom (or atoms) in a molecular entity and a nucleophilic region in another, or the same, molecular entity [81]. The same underlying definition applies to a pnictogen bond, with the terms “tetrel bond” and “tetrel atom” replaced by the terms “pnictogen bond” and “tetrel atom”, respectively. Both the pnictogen and tetrel bonds are linked, with a bent ∠Pb–X···Pb angle between the two ion pairs forming the [CH_3_NH_3_^+^•PbI_3_^−^]_2_ dimer. Although these were obtained from gas phase calculations, a very similar geometric feature was found in the low-temperature orthorhombic structure of the CH_3_NH_3_PbX_3_ perovskites (X = Cl, Br) (Figure 6e–g); however, the X–Pb bonds in the latter are nearly equivalent as a result of crystal packing forces.

**Figure 7:**
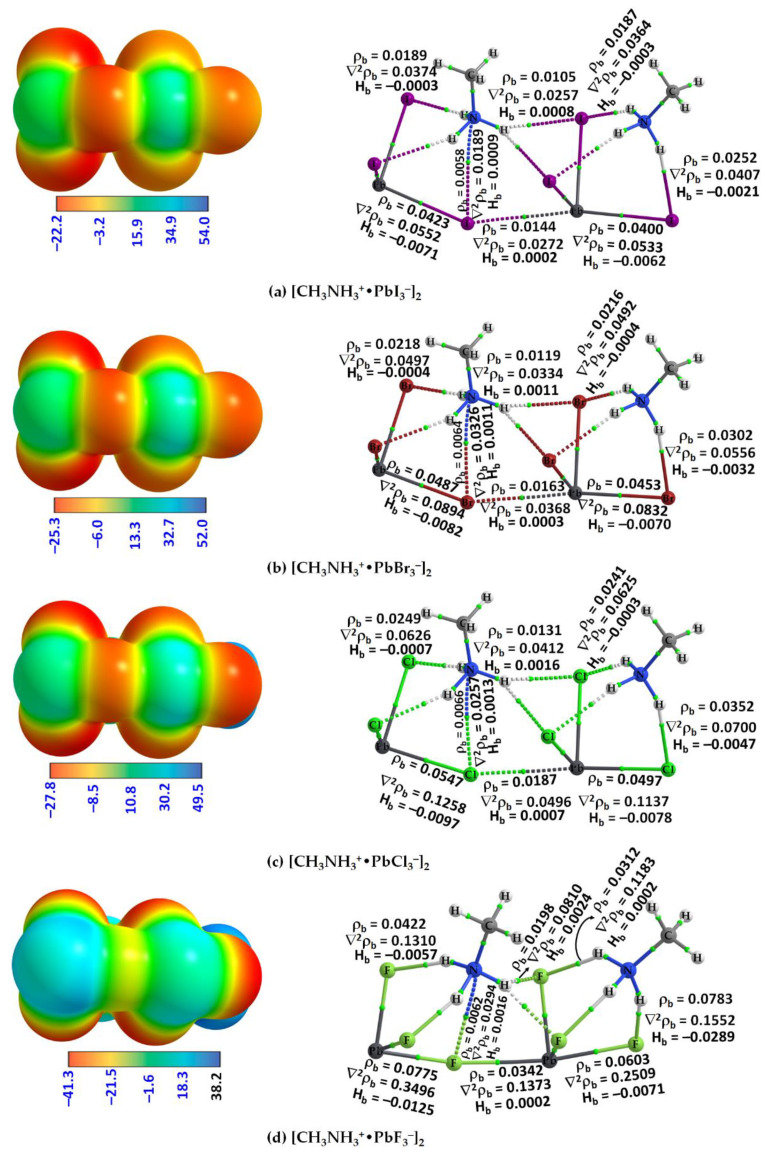
(**Left**) [ωB97X-D/def2-TZVPPD] computed 0.001 a.u. mapped electrostatic potential on the molecular surfaces of (**a**) [CH_3_NH_3_^+^•PbI_3_^−^]_2_; (**b**) [CH_3_NH_3_^+^•PbBr_3_^−^]_2_; (**c**) [CH_3_NH_3_^+^•PbCl_3_^−^]_2_; and (**d**) [CH_3_NH_3_^+^•PbF_3_^−^]_2_. (**Right**) QTAIM-based molecular graphs of the corresponding systems, respectively. (The bond critical points between atomic basins are shown as tiny spheres in green, and bond paths in atom colors.) Values on the color bar are in kcal mol^−1^, with the surface involving the fragment Pb–X···Pb–X facing the reader.

A question that arises is why the [PbI_3_^−^] anions are bonded to each other in the crystal, forming an infinite array of [PbX_6_^4−^]_∞_ octahedra that form cage-like structures to host the organic cations, even though the anions are expected to coulombically repel each other. An immediate answer to this is probably the presence of CH_3_NH_3_^+^ that locally polarizes the potential on the electrostatic surface of the tetrel atom in [PbI_3_^−^] when in close proximity, driven by an extensive number of double-charge-assisted N–H···X and C–H···X hydrogen bonds that appear between them. As the surface of the tetrel atom in the ion pair [CH_3_NH_3_^+^•TtX_3_^−^] is highly electrophilic, featuring three positive σ-holes on Tt (Tt = Pb and Sn), it is capable of accepting electrons simultaneously from three halogens of three interacting ion pairs [CH_3_NH_3_^+^•TtI_3_^−^] at equilibrium, forming the [TtX_6_^4−^] octahedra in the crystalline phase. QTAIM calculations suggest there is an appreciable transfer of charge between the organic and inorganic ions, facilitating the formation of the ion pair, and this is true in all three conformations investigated for [CH_3_NH_3_^+^•TtX_3_^−^], a feature that also occurs between the ion pairs responsible for the dimers. When the same process of assembly continues with [CH_3_NH_3_^+^•PbX_3_^−^] ion pairs, the formation of CH_3_NH_3_TtX_3_ perovskite in the solid state is the likely consequence, a result of interplay between the σ-hole-centered tetrel bonds and other non-covalent interactions. The MESP plots that provide evidence of the formation of X–Tt···X tetrel bonds (dotted lines) between two ion pairs, leading to the formation of [CH_3_NH_3_^+^•PbX_3_^−^]_2_ dimers, are shown in Figure 7a–d (left). They show that the positive σ-hole on Pb in an ion pair is in coulombic attraction with the negative halide of the anion with which it interacts.

As shown in Figure 6a–d, the organic cation in the [CH_3_NH_3_^+^•PbX_3_^−^] ion pair on the left connects with another ion pair on the right through N–H···X hydrogen bonds and X–Tt···X tetrel bonds. The former are equivalent in a given ion pair, and become shorter as the size of the halogen decreases from I through to F. Based on the bond distances, it is clear that the hydrogen bonds are stronger in [CH_3_NH_3_^+^•PbF_3_^−^] and weaker in [CH_3_NH_3_^+^•PbI_3_^−^]. This might be expected since the lighter halogens are more electronegative and hence able to form stronger hydrogen bonds. Partial halogen transfer from [PbF_3_^−^] on the right ion pair [CH_3_NH_3_^+^•PbF_3_^−^] towards the ammonium H atom is evident in the [CH_3_NH_3_^+^•PbF_3_^−^]_2_ dimer (Figure 6d); this may indicate that the formation of the perovskite CH_3_NH_3_PbF_3_ is unlikely in the solid state. The result also suggests that the formation of a different type of ion pair, methylammonium fluoride ([CH_3_NH_3_^+^•F^−^]), is a likely consequence when more than two units of the [CH_3_NH_3_^+^•PbF_3_^−^] ion pair are in close proximity. The geometric arrangement between them would hinder the formation CH_3_NH_3_PbF_3_ perovskite in the crystalline phase and might explain why CH_3_NH_3_PbF_3_ is unknown in the solid state.

The X–Tt···X tetrel bonds formed between the ion pairs in the dimers at equilibrium are all less than the sum of the van der Waals (vdW) radii of Tt and X; for instance, the tetrel bond distances of 3.582, 3.351, 3.163 and 2.530 Å in [CH_3_NH_3_^+^•PbI_3_^−^]_2_ (Figure 6a), [CH_3_NH_3_^+^•PbBr_3_^−^]_2_ (Figure 6b), [CH_3_NH_3_^+^•TPbCl_3_^−^]_2_ (Figure 6c) and [CH_3_NH_3_^+^•PbF_3_^−^]_2_ (Figure 6d) are much less than the vdW radii sum 4.64 (Pb + I), 4.46 (Pb + Br), 4.42 (Pb + Cl) and 3.50 Å (Pb + F) [104], respectively. Moreover, they are quasi-linear, (∠X–Pb···X values between 168° and 176°). This signature satisfies characteristic features *d* and *f* and Note 4 of tetrel bonds discussed in a recently suggested definition of a tetrel bond [81].

The geometries of the isolated ion pair, [CH_3_NH_3_^+^•TtX_3_^−^], and its dimer, [CH_3_NH_3_^+^•TtX_3_^−^]_2_, and the intermolecular interactions in them are not identical. A major rearrangement of the monomers occurred on dimer formation. In particular, the organic cation shared between the two inorganic anions rearranged in a manner so as to maximize its non-covalent interactions with the halides of the two inorganic moieties (Figure 6), while the organic cation on the right retains its shape as observed in the isolated ion pair itself (cf. Figure 2). The physical arrangement between the ions in the former (left) part of the dimer resembles the geometry of crystalline tetrel halide perovskites (Figure 6e–g) in the low-temperature orthorhombic phase. The corner-shared [TtX_6_^4−^] octahedra in these structures are tilted along the crystallographic *a*–*c*-axes. The tilting can be gauged from the ∠Pb–X···Pb angle between a pair of octahedra that are located at corners of a significantly distorted cube.

The tilt angle is smallest in the [CH_3_NH_3_^+^•PbI_3_^−^]_2_ dimer (∠Pb–I···Pb = 146.4°) and increases as the size of the halogen in [CH_3_NH_3_^+^•PbX_3_^−^]_2_ decreases from Br through Cl to F (∠Pb–Br···Pb = 151.7°; ∠Pb–Cl···Pb = 156.2°; ∠Pb–F···Pb = 168.7°). Although this angle is obtained from a linearly arranged structure in the gas phase, it reflects the same feature observed in the crystalline phase. For instance, the Cl–Pb–Cl angles are 155.1°, 160.9° and 163.9° in the low-temperature (100 K) orthorhombic (o) phase of MAPbCl_3_ [111] (ICSD ref: 241415) along the crystallographic *a-*, *b-* and *c*-directions, respectively (two are shown in Figure 6g). In the case of (120 K) o-MAPbBr_3_ [112] (ICSD ref: 268782), the corresponding angles are 157.7°, 169.6° and 157.7°, respectively (two are shown in Figure 6f). In the *Pnma* structure of (100 K) o-MAPbI_3_ [113] (ICSD ref: 428898), the tilt of the octahedra is such that the Pb–I–Pb angles (Figure 6e) are Pb–I–Pb = 161.94(16)° (along *b*-axis) and Pb–I–Pb = 150.75(12)° (along the *a-* and *c*-axes) respectively. The average tilt angle, ∠Pb–X–Pb, in o-MAPbCl_3_, o-MAPbBr_3_ and o-MAPbI_3_ is 160.0°, 161.4° and 154.5°, respectively, in reasonable agreement with the tilt angles of the gas phase dimers. Geometric details of [CH_3_NH_3_^+^•TtX_3_^−^]_2_ (Tt = Sn, Ge, Si; X = F, Cl, Br, I) dimers are given in Figures S7 and S8 of the ESI.

It has been shown that to improve the efficiency of perovskite solar cells, it is necessary to tune the degree of octahedral tilting of the halide framework, which affects the optical band gap and the effective mass of the charge carriers [75,114]. The steric effects dominate the magnitude of the tilt in inorganic halides, while hydrogen bonding between the organic cation and the halide frame plays an important role in hybrids, and tuning the degree of hydrogen bonding can be used as an additional control parameter to optimize the photoelectric conversion properties of the perovskites [75]. In the absence of hydrogen bonding, the octahedra in the tetrel halide perovskites do not tilt at all, a view which is in disagreement with our results discussed above and elsewhere [22]. In the case of all-inorganic alkali tetrel halide perovskites, the in-phase tilting provides a better arrangement of the larger bromide and iodide anions, which minimizes the electrostatic interactions, improves the bond valence of the A-site cations and enhances the covalency between the A-site metal and Br^−^ or I^−^ ions [115].

The chemical bonding shown in the left portion of the [CH_3_NH_3_^+^•PbX_3_^−^]_2_ dimer (Figure 6a–c), which includes N–H···X hydrogen bonds, a C–N···X pnictogen bond and an X–Pb···X tetrel bond, is very similar to that found in crystals of tetrel halide perovskites. A major difference between them is that the gas phase structures do not have hydrogen bonds formed by the methyl group of the organic cation; these can evolve if the [CH_3_NH_3_^+^•PbX_3_^−^]_2_ dimer is surrounded with additional ion pairs in all three directions. Likewise, the C–N···X pnictogen bond apparent in the left portion is absent on the right of the [CH_3_NH_3_^+^•PbX_3_^−^]_2_ dimer. An extended array of [CH_3_NH_3_^+^•PbX_3_^−^] ion pairs to the right of the dimer will enable the terminal organic cation to rearrange in a manner as on the left so the terminal hydrogen H atom of the ammonium group of the organic cation on the right can engage in the formation of N–H···X bonding interactions with the halogen atoms of a third ion pair; the N site of the same cation can also engage with the tetrel-bonded X site to form a C–N···X pnictogen bond. These intermolecular interactions were confirmed by a QTAIM analysis, see Figure 7 (right). The analysis shows that there is variability in the character of the N–H···X hydrogen bonding interactions in the gas phase geometries, characterized by the topological properties of the charge density.

The QTAIM results suggest that the strength of the C–N···X pnictogen bond increases with an increase in the size of the halogen in [CH_3_NH_3_^+^•TtX_3_^−^]_2_. For instance, *ρ*_b_ (∇^2^*ρ*_b_) [*H*_b_] values at the N···X bcps were 0.0070 (0.0231) [0.0010], 0.0080 (0.0293) [0.0012], 0.0086 (0.0339) [0.0015] and 0.0094 (0.0463) [0.0023] a.u. for [CH_3_NH_3_^+^•SnI_3_^−^]_2_, [CH_3_NH_3_^+^•SnBr_3_^−^]_2_, [CH_3_NH_3_^+^•SnCl_3_^−^]_2_ and [CH_3_NH_3_^+^•SnF_3_^−^]_2_, respectively. The positive sign of both ∇^2^*ρ*_b_ and *H*_b_ indicates that the pnictogen bond is of the closed-shell type, and its magnitude signifies that the close contact is more ionic in nature in [CH_3_NH_3_^+^•SnF_3_^−^]_2_ than in [CH_3_NH_3_^+^•SnI_3_^−^]_2_. The trend in the charge density properties is in agreement with the geometric features of the pnictogen bond, viz. bond distance *r*(N···X) (and bond angle (∠C–N···X)): 3.634 (174.3°), 3.406 (172.1°), 3.261 (170.2°) and 2.870 Å (172.6°) in [CH_3_NH_3_^+^•SnI_3_^−^]_2_, [CH_3_NH_3_^+^•SnBr_3_^−^]_2_, [CH_3_NH_3_^+^•SnCl_3_^−^]_2_ and [CH_3_NH_3_^+^•SnF_3_^−^]_2_, respectively. These are very similar to values found for the [CH_3_NH_3_^+^•TtX_3_^−^]_2_ dimers, viz. the corresponding values in [CH_3_NH_3_^+^•PbI_3_^−^]_2_, [CH_3_NH_3_^+^•PbBr_3_^−^]_2_, [CH_3_NH_3_^+^•PbCl_3_^−^]_2_ and [CH_3_NH_3_^+^•PbF_3_^−^]_2_ were 3.747 (173.8°), 3.537 (170.7°), 3.396 (171.5°) and 3.069 Å (174.1°), respectively. They were 3.582 (172.5°), 3.350 (170.4°), 3.211 (167.1°) and 2.761 Å (178.5°) for [CH_3_NH_3_^+^•GeI_3_^−^]_2_, [CH_3_NH_3_^+^•GeBr_3_^−^]_2_, [CH_3_NH_3_^+^•GeCl_3_^−^]_2_ and [CH_3_NH_3_^+^•GeF_3_^−^]_2_, respectively, and 3.535 (172.6°), 3.311 (170.5°) and 3.169 Å (164.4°), for [CH_3_NH_3_^+^•SiI_3_^−^]_2_, [CH_3_NH_3_^+^•SiBr_3_^−^]_2_ and [CH_3_NH_3_^+^•SiCl_3_^−^]_2_, respectively.

The relatively longer pnictogen bonds in the [CH_3_NH_3_^+^•SiX_3_^−^]_2_ dimers are a result of increasing repulsion between the interacting atomic basins (Si and X) when two ion pairs are in close proximity. The stability of these Si-based dimers is governed by N–H···X hydrogen bonds formed by the organic cation common to both the interacting ion pairs. Details of the topological charge density properties associated with the pnictogen bond are given in Table 3, and the molecular graphs of the dimers with Tt = Sn, Ge and Si are shown in Figures S9a–d, 10a–d and 11a–d of the ESI, respectively. Formation of a dimer is accompanied by an induction of weakly electrophilic σ-holes on the surface of the coordinately bound Si when X = I, Cl and Br but not when X = F, explaining why there is a weak Si···X tetrel bond in the first three dimers but not in [CH_3_NH_3_^+^•SiF_3_^−^]_2_ (see Figures S8d and S11d of the ESI). In the case of the latter, the development of electrophilic sites on Si is seen only in the equilibrium structure of the system; induction of the electrophilic sites does not occur during the course of the interaction between the two ion pairs because of coulombic repulsion between entirely negative Si and F sites in the two interacting ion pairs. The feature is shown in Figure S12 of the ESI for [CH_3_NH_3_^+^•SiX_3_^−^]_2_ (X = F, Cl, Br, I).

In the case of the [CH_3_NH_3_^+^•SiF_3_^−^]_2_ dimer, the Si center in the [CH_3_NH_3_^+^•SiF_3_^−^] ion pair on the left of Figure S8d of the ESI acts as a hydrogen bond acceptor for an ammonium H of the organic cation in the second ion pair on the right when they are in close proximity. The interaction energy of the Si···H hydrogen bond was calculated to be –14.35 kcal mol^−1^ and this does not involve any secondary interactions between the two ion pairs. Each of the C–N···X (X = F, Cl, Br, I) pnictogen bonds found in the four other [CH_3_NH_3_^+^•SiX_3_^−^]_2_ dimers was stronger than the Si···X tetrel bonds found in [CH_3_NH_3_^+^•SiX_3_^−^]_2_ (X = I, Br, Cl), which can be inferred from the *ρ*_b_ values at the bcps shown in Figure S11a–d of the ESI and in Table 3. These are characterized by a positive value of ∇^2^*ρ*_b_ and *H*_b_, indicative of closed-shell interactions; these are also present in the orthorhombic crystals of MAPbX_3_ (see Figure S13 of the ESI for MAPbI_3_ and MAPbBr_3_) and are discussed elsewhere [22,23]. The Si···H hydrogen bond in [CH_3_NH_3_^+^•SiF_3_^−^]_2_ has a covalent character since ∇^2^*ρ*_b_ > 0 and *H*_b_ < 0) (see Figure S11d and Table 3 for values).

The uncorrected and BSSE-corrected interaction energies, Δ*E* and Δ*E(*BSSE*)*, respectively, of all the [CH_3_NH_3_^+^•TtX_3_^−^]_2_ (Tt = Pb, Sn, Ge, Si; X = I, Br, Cl, I) dimers examined are given in Table 4. The interaction energy holding the two ion pairs together in the dimers decreases for a given type of halogen derivative in [CH_3_NH_3_^+^•TtX_3_^−^]_2_ (except [CH_3_NH_3_^+^•SiF_3_^−^]_2_). This is attributed to the polarizability of the Tt derivative that decreases as the size of the tetrel atom decreases from Pb through to Si. For instance, the BSSE-corrected interaction energies, Δ*E*(BSSE), are −16.83, −13.51, −11.66 and −9.92 kcal mol^−1^ for [CH_3_NH_3_^+^•PbI_3_^−^]_2_, [CH_3_NH_3_^+^•SnI_3_^−^]_2_, [CH_3_NH_3_^+^•GeI_3_^−^]_2_ and [CH_3_NH_3_^+^•SiI_3_^−^]_2_, respectively. The decrease in the interaction energy in the series is also attributed to the weakening of the tetrel bond between the ion pairs (see Figure S7 of the ESI, for example, for [CH_3_NH_3_^+^•SnX_3_^−^]_2_, as well as discussion on [CH_3_NH_3_^+^•PbX_3_^−^]_2_ and [CH_3_NH_3_^+^•GeX_3_^−^]_2_). It should be noted that the interaction energy is not due solely to the tetrel bond but also due to several hydrogen bonds that are the key forces holding the ion pairs together in the equilibrium geometry of each of the dimers.

We did not observe any systematic trend in Δ*E*(BSSE) values for any given Tt in [CH_3_NH_3_^+^•TtX_3_^−^]_2_. However, our results show that Δ*E*(BSSE) increases as the size of the halogen derivative decreases from I through to F. The trend does not correlate with the strength of the σ-hole on the surface of the Tt atom in the ion pairs. For instance, the σ-holes on Pb along the X–Pb bond extensions in [I_3_Pb···NH_3_CH_3_], [Br_3_Pb···NH_3_CH_3_], [Cl_3_Pb···NH_3_CH_3_] and [F_3_Pb···NH_3_CH_3_] are 26.1, 26.8, 26.1 and 22.8 kcal mol^−1^, respectively. Accordingly, the same trend in the interaction energy could be expected for [CH_3_NH_3_^+^•PbI_3_^−^]_2_, [CH_3_NH_3_^+^•PbBr_3_^−^]_2_, [CH_3_NH_3_^+^•PbCl_3_^−^]_2_ and [CH_3_NH_3_^+^•PbF_3_^−^]_2_, respectively, yet the observed trend follows the order: [CH_3_NH_3_^+^•PbI_3_^−^]_2_ < [CH_3_NH_3_^+^•PbBr_3_^−^]_2_ < [CH_3_NH_3_^+^•PbCl_3_^−^]_2_ < [CH_3_NH_3_^+^•PbF_3_^−^]_2_. The increase in the interaction energy with the decrease in the size of the halogen in [CH_3_NH_3_^+^•PbX_3_^−^]_2_ is clearly a result of strong hydrogen bonding between the ion pairs that play a vital role in stabilizing the tetrel bonds. It is worth mentioning that the trend in the decrease in the interaction energy correlates with the decreasing strength of the σ-hole on Ge in [X_3_Ge···NH_3_CH_3_] for [CH_3_NH_3_^+^•GeX_3_^−^]_2_ series, although this was not so for the [CH_3_NH_3_^+^•SnX_3_^−^]_2_ series. We have not performed similar calculations with other theoretical methods, but our MP2 level calculations for the X_3_Pb^−^···MA (X = I, Br, Cl, F, and MA = NH_3_CH_3_^+^) ion pair series, in conjunction with the def2-TZVPPD and aug-cc-pVTZ basis set, have produced a similar trend (Br > I > Cl > F) for the strength of the σ-hole on Pb along the X–Pb bond extensions (see Tables S1 and S2).

## 3. Discussion

The application of the current state-of-the-art theoretical methods has revealed various types of intermolecular (interionic) interactions between organic and inorganic ions, leading to the formation of molecular methylammonium tetrahalide perovskite ion pairs in the gas phase. Of the ion pairs investigated, for any given halogen and tetrel derivative, the conformer with the amine group facing towards the triangular face of the inorganic anion was found to be the most stable (i.e., Conf. 1). This is similar to the geometry observed in the solid state with the organic cation oriented along the (111) direction. The other two conformers (Conf. 2 and 3) known (and extracted) from lead halide perovskite crystal in the cubic phase were found to be the second- and first-order saddle-point structures, respectively.

The conclusion that emerged from combined second-order hyperconjugative charge transfer delocalization and charge-density based IGM-*δg^inter^* analyses is that the most stable ion pair for each Tt series [CH_3_NH_3_^+^•TtX_3_^−^] (Tt = Pb, Sn, Ge, Si; X = I, Br, Cl, F), with the organic cation along the (111) direction as observed in the solid state, is not entirely stabilized by N–H···X hydrogen bonds but also partially by a C–N···I pnictogen bond. The latter interaction cannot be readily identifiable by just looking at the geometry of the ion pair yet can be revealed at a very low isovalue around 0.008 a.u. using an IGM-*δg^inter^* analysis. This is not unexpected given that anti-bonding σ*(N–C) is an electron-accepting orbital and hence capable of accepting electrons from the lone pair orbital of coordinate I atoms of the inorganic anion when they are in close proximity.

The MESP analysis of the ion pairs led to the identification and subsequent characterization of electrophilic and/or nucleophilic σ-holes on the surface of the tetrel derivative in the ion pairs examined. The strength of the σ-hole was shown to vary with the size of the halogen derivative for any given tetrel atom in the ion pair. There were three such equivalent σ-holes on the electrostatic surface of Tt in each ion pair when the methyl or ammonium end of the organic cation faced towards the triangular face of the inorganic cation. They were inequivalent when the organic cation was in a sitting orientation (viz. (011) orientation in the cubic lattice in the solid state) (Conf. 3). Our calculations suggest that the effect of electrostatic polarization (and/or charge transfer) of the organic cation plays a crucial role in transforming the negative potential to positive on the surface of the tetrel derivative in TtX_3_^−^ in the majority of the ion pairs, and hence three electrophilic σ-holes appeared, especially when Tt = Pb, Sn (except [F_3_Sn···CH_3_NH_3_]).

Except for the three conformations of F_3_Ge···MA and X_3_Ge···CH_3_NH_3_ (X = I, Br, Cl), the surfaces of Ge in the remaining ion pairs of the same series were electrophilic, which suggests that the methyl end is relatively less effective in polarizing the GeX_3_^−^ anion compared to when the ammonium head faces the triangular halide face of the anion ion in the ion pair. Except for a few cases (viz. I_3_Si···NH_3_CH_3_ and Br_3_Si···NH_3_CH_3_), the organic cation was unable to polarize the electron charge density on the surface of Si in [X_3_Si···MA]), thus nucleophilic σ-holes developed on its electrostatic surface.

Gas phase exploration of the dimers of each of the ion pairs investigated has enabled us to reveal why the inorganic anion is capable of forming corner-shared TtX_6_^4−^ octahedra in the solid state, especially when X = I, Br and Cl and Tt = Pb, Sn and Ge. In particular, our results led to the conclusion that the coulombic attraction between the monovalent anions TtX_3_^−^ leading to the TtX_6_^4−^ octahedra in an infinite array in the crystalline material is driven by (X_3_^−^)Tt···X(TtX_2_^−^) tetrel bonds in the presence of the organic cation MA. Three such tetrel bonds can be formed by each Tt center in each TtX_3_^−^ anion when each ion pair is surrounded by three identical ion pairs, forming the corner-shared TtX_6_^4−^ octahedra, in which each octahedron occupies a corner of a regular cube in the high-temperature phase of the system. Although our calculation was limited to the gas phase and dimers of some representative ion pairs, our results have provided evidence of possible tilting of the TtX_6_^4−^ octahedra observed in the low-temperature orthorhombic phase of MATtX_3_ (X = I, Br, Cl; Tt = Pb, Sn), driven by different types of intermolecular interactions. In particular, the electronic structures of the dimer models, in combination with the QTAIM results, suggested that C–N···X pnictogen bonds play a critical role, in addition to the MA(X_3_^−^)Tt···X(TtX_2_^−^)MA tetrel bonds and N–H···X and C–H···X hydrogen bonds, which interact in determining the octahedral tilting. The formation of (C)N···X pnictogen bonds is an inherent feature of the organic–inorganic tetrel halide perovskites and is present in all the 16 dimers investigated, whether or not the geometry of the dimer mimics a part of the tetrel halide perovskite structure in the crystalline phase. Therefore, it would be misleading to attribute the octahedral tilting feature in methylammonium tetrel halide perovskites to just the N–H···X hydrogen bonds. It should be borne in mind that the intermolecular interactions between the ions leading to the formation of an ion pair are charge-assisted, whereas those responsible for the interaction between ion pairs are not, given that each ion pair is a neutral system and the assembly between the ion pairs in dimers or extended systems is driven by ordinary medium-to-strong non-covalent interactions.

Our investigation has also shed light on why MATtX_3_ (X = F, Br, Cl; Tt = Ge, Si) perovskites are not known in the crystalline phase. The failure to synthesize these systems was speculated on when exploring the MESP of the ion pairs of the corresponding systems. The σ-holes on the Tt sites in these systems were weakly positive (or even nucleophilic) and are unlikely to be able to engage appreciably in attractive (coulombic) intermolecular interactions with the halides of a neighboring ion pair. While [CH_3_NH_3_^+^•TtX_3_^−^]_2_ (X = Cl, Br, I; Tt = Si) perovskite dimers are stable in the gas phase as result of N–H···X strong hydrogen bonding, the MASiX_3_ perovskites are not likely to be formed in the crystalline phase because the Si···X tetrel bonds between the ion pairs are very weak; they are not only secondary interactions but also driven by N–H···X hydrogen bonds formed between the ion pairs when in close proximity.

## 4. Materials and Methods

The DFT-*ω*B97X-D [87] calculations, in combination with the basis set def2-TZVPPD, were performed to fully relax the geometry of 46 ion pairs and their binary complexes; a binary complex refers to the dimer of an ion pair. The density functional uses a version of Grimme’s D2 dispersion model [116], as implemented in Gaussian 16 [110]; the basis set is available in the EMSL basis set exchange library [117,118]. Default convergence criteria (tight SCF and ultrafine integration grid) were used. A harmonic frequency calculation was performed in each case. The identified local minima and saddle points are discussed in the Results section. Second-order Møller–Plesset perturbation theory (MP2) [119,120] calculations were also performed on a few systems to demonstrate the reliability of the results obtained with DFT. Although the theoretical methods discussed above were employed on the chemical systems in the gas phase, the results shown in Figure 1 were obtained using periodic boundary calculations, in which the popular PBEsol [121] functional implemented in VASP 5.4 [96,97,98,99,100] was utilized. This was carried out to illustrate the geometric aspects of analogues of the ion pairs responsible for the crystalline phase.

The MESP model [89,90,91,92] generates two physical descriptors that are the local minima and maxima of the potential (*V_S,min_* and *V_S,max_*, respectively [71,90,122,123,124,125,126,127,128]) when mapped on an isoelectronic density envelope of a molecule. The positive/negative signs of *V_S,min_* and *V_S,max_* ([*V_S,min_* > 0 and *V_S,max_* > 0]/[*V_S,min_* < 0 and *V_S,max_* < 0]) represent the electrophilicity/nucleophilicity of a particular region on the electrostatic surface of the molecular domain. The magnitude of *V_S,min_* or *V_S,max_* determines the strength of the potential and correlates linearly with the strength of the intermolecular interactions. The usefulness of the model has been demonstrated in a number of studies. Following a prior recommendation [129,130], the 0.001 a.u. isoelectronic density was used to map the potential for all cases. However, we have also shown that the choice of this envelope can be misleading in systems where the anion contains a more electronegative (and hence less polarizable) tetrel derivative and the less acidic portion of the organic cation faces the anion to form an ion pair. In this case, the use of a higher-value isodensity envelope is necessary [125,131,132,133,134,135,136]. A σ-hole on the surface of Tt along the outermost extension of the R–Tt covalent/coordinate σ-bond in a molecule was identified when the most local potential associated with it is positive (*V_S,max_* > 0); R is the remaining part of the molecule [81].

QTAIM [88] relies on the zero-flux boundary condition and the bond path topology recovers the connectivity between covalently bonded atoms that make up the ion pair or dimers examined. Analysis of the reduced charge density-based isosurfaces was performed using the actual density computed within the framework of IGM-δg*^inter^* [93,94]. Software such as AIMAll [137] and Multiwfn [138,139], together with VMD [140], were used for QTAIM, MESP and IGM-δg*^inter^* analyses and drawing of MESP and IGM-δg*^inter^* graphs.

The uncorrected and Basis Set Superposition Error (BSSE)-corrected interaction energies (Δ*E* and Δ*E*(BSSE), respectively) were calculated using Equations (1) and (2). *E*_T_ in Equation (1) and *E*(BSSE) in Equation (2) are the electronic total energy of respective species and the error in total electronic energy due to the effect of the basis set superposition accounted for by the counterpoise procedure of Boys and Bernardi [109], respectively. The total electronic energy of the individual ion (or ion pair) was calculated using the energy-minimized geometry of the ion pair (or binary complex), which was used for the calculation of the interaction energy.

Δ*E* (dimer) = *E*_T_(dimer) − [*E*_T_(monomer 1) + *E*_T_(monomer 2)] (1)

Δ*E*(BSSE) = Δ*E*(dimer) + *E*(BSSE) (2)

## Figures and Tables

**Table 1:** Selected [*ω*B97X-D/def2-TZVPPD] computed 0.001 a.u. isoelectronic density envelope mapped potential maxima and minima (kcal mol^−1^) on the electrostatic surfaces of ion pairs X_3_Tt^−^···MA (X = I, Br, Cl, F; Tt = Pb, Sn, Ge, Si; MA = NH_3_CH_3_^+^). The 0.0015 a.u. isoelectronic density envelope mapped potentials are given in parentheses for selected systems.

Ion Pairs	*V_S,max_*(X–Tt)	*V_S,max_*(X–Tt)	*V_S,max_*(X–Tt)	*V_S,min_*(Tt)	*V_S,max_*(N-C)/*V_S,max_*(C-N)	*V_S,min_*(X)	*V_S,min_*(X)
I_3_Pb···NH_3_CH_3_	26.1	26.1	26.1	22.2	38.2	−18.0	
I_3_Pb···CH_3_NH_3_	11.9	11.9	11.9	9.6	90.4	−29.9	
I_3_Pb···H_3_NCH_3_	23.4	23.4	19.4	18.3	---	−23.1	−17.4
Br_3_Pb···NH_3_CH_3_	26.6	26.6	26.6	22.2	36.4	−21.2	
Br_3_Pb···CH_3_NH_3_	10.4	10.4	10.4	7.7	88.3	−34.1	
Br_3_Pb···H_3_NCH_3_	23.0	23.0	19.1	17.4	---	−27.2	−21.5
Cl_3_Pb···NH_3_CH_3_	26.1	26.1	26.1	21.0	34.9	−23.8	
Cl_3_Pb···CH_3_NH_3_	8.3	8.3	8.3	5.1	86.6	−37.0	
Cl_3_Pb···H_3_NCH_3_	21.8	21.8	17.9	15.5	---	−30.5	−26.9
F_3_Pb···NH_3_CH_3_	22.8	22.8	22.8	14.8	27.4	−37.6	
F_3_Pb···CH_3_NH_3_	−0.4 (4.1)	−0.4 (4.1)	−0.4 (4.1)	−5.7 (−4.2)	79.6 (83.8)	−53.1 (−57.0)	
F_3_Pb···H_3_NCH_3_	16.1	16.1	12.5	6.8	---	−39.2	−47.5
I_3_Sn···NH_3_CH_3_	15.6	15.6	15.6	5.3	39.5	−16.9	
I_3_Sn···CH_3_NH_3_	0.5	0.5	0.5	−7.9	91.4	−30.0	
I_3_Sn···H_3_NCH_3_	12.5	12.5	9.5	1.6	---	−22.2	−17.2
Br_3_Sn···NH_3_CH_3_	15.5	15.5	15.5	3.8	38.2	−19.8	
Br_3_Sn···CH_3_NH_3_	−1.6 (3.6)	−1.6 (3.6)	−1.6 (3.6)	−11.1 (−10.1)	89.8 (94.4)	−33.8 (−34.7)	
Br_3_Sn···H_3_NCH_3_	11.5	11.5	8.6	−0.7	---	−25.8	−20.9
Cl_3_Sn···NH_3_CH_3_	14.6	14.6	14.6	1.7	37.2	−22.1	
Cl_3_Sn···CH_3_NH_3_	−4.0 (1.1)	−4.0 (1.1)	−4.0 (1.1)	−14.8 (−14.2)	88.5 (93.0)	−35.8 (−37.8)	
Cl_3_Sn···H_3_NCH_3_	10.1	10.1	7.2	−3.6	---	−28.7	---
F_3_Sn···NH_3_CH_3_	11.5	11.5	11.5	−5.4	31.6	−34.6	
F_3_Sn···CH_3_NH_3_	−11.7 (−6.9)	−11.7 (−6.9)	−11.7 (−6.9)	−25.8 (−26.4)	82.8 (---)	−50.8 (−54.4)	
F_3_Sn···H_3_NCH_3_	5.3	5.3	3.3	−12.3		−36.3	−44.0
I_3_Ge···NH_3_CH_3_	7.0	7.0	7.0	−3.0	40.6	−17.3	
I_3_Ge···CH_3_NH_3_	−8.2 (−3.8)	−8.2 (−3.8)	−8.2 (−3.8)	−16.0 (−15.3)	92.5 (97.1)	−30.9 (−31.1)	
I_3_Ge···H_3_NCH_3_	4.2	4.2	1.4	−6.1	---	−22.2	−17.8
Br_3_Ge···NH_3_CH_3_	6.1	6.1	6.1	−5.5	39.7	−19.9	
Br_3_Ge···CH_3_NH_3_	−11.3	−11.3	−11.3	−20.7	90.9	−35.4	
Br_3_Ge···H_3_NCH_3_	2.4 (7.4)	2.4 (7.4)	−0.3 (4.3)	−9.6 (−8.9)	---	−25.4 (−26.4)	−21.4 (−22.4)
Cl_3_Ge···NH_3_CH_3_	4.8	4.8	4.8	−8.7	39.0	−21.7	
Cl_3_Ge···CH_3_NH_3_	−14.2	−14.2	−14.2	−25.3	89.7	−37.9	
Cl_3_Ge···H_3_NCH_3_	0.6 (5.9)	0.6 (5.9)	−1.8 (2.7)	−13.4 (−13.1)	---	−27.6 (−29.0)	−24.1 (−25.1)
F_3_Ge···NH_3_CH_3_	−0.3 (4.7)	−0.3 (4.7)	−0.3 (4.7)	−19.2 (−19.9)	35.4 (38.0)	−33.1 (−35.5)	
F_3_Ge···CH_3_NH_3_	−23.8	−23.8	−23.8	−39.5	85.2	−50.3	
F_3_Ge···H_3_NCH_3_	−6.1	−6.1	−6.7	−25.1	---	−41.1	−35.3
I_3_Si···NH_3_CH_3_	−0.8 (3.3)	−0.8 (3.3)	−0.8 (3.3)	−15.8 (−16.0)	41.8 (44.7)	−16.6 (−16.7)	
I_3_Si···CH_3_NH_3_	−16.3	−16.3	−16.3	−29.1	93.4	−30.6	
Br_3_Si···NH_3_CH_3_	−3.1 (1.3)	−3.1 (1.3)	−3.1 (1.3)	−20.5 (−21.3)	41.3 (44.2)	−19.2 (−19.7)	
Br_3_Si···CH_3_NH_3_	−20.9	−20.9	−20.9	−35.7	92.3	−35.0	
Cl_3_Si···NH_3_CH_3_	−5.3 (−1.0)	−5.3 (−1.0)	−5.3 (−1.0)	−25.5 (−26.7)	41.3 (44.2)	−21.0 (−21.7)	
Cl_3_Si···CH_3_NH_3_	−24.6	−24.6	−24.6	−41.9	91.7	−37.6	
Cl_3_Si···H_3_NCH_3_	−9.8	−9.8	−10.8	−29.8	---	−24.5	−27.5
F_3_Si···NH_3_CH_3_	−12.6	−12.6	−12.6	−41.2	40.8	−32.1	
F_3_Si···CH_3_NH_3_	−35.0	−35.0	−35.0	−60.0	89.6	−50.6	
F_3_Si···H_3_NCH_3_	−17.70	−17.70	−15.0	−45.8	---	−34.2	−39.4

**Table 2:** The uncorrected and BSSE-corrected interaction energies, Δ*E* and Δ*E(*BSSE*)*, respectively, of methylammonium tetrel halide perovskite ion pairs, obtained with [*ω*B97X-D/def2-TZVPPD]. Values in kcal mol^−1^.

System	Interaction Type	Δ*E*	Δ*E*(BSSE)
I_3_Pb···NH_3_CH_3_	I···H(N)	−104.95	−104.85
I_3_Pb···CH_3_NH_3_	I···H(C)	−76.42	−76.33
I_3_Pb···H_3_NCH_3_	I···H(N), I···H(C)	−97.64	−97.53
Br_3_Pb···NH_3_CH_3_	Br···H(N)	−111.04	−110.66
Br_3_Pb···CH_3_NH_3_	Br···H(C)	−80.05	−79.77
Br_3_Pb···H_3_NCH_3_	Br···H(N), Br···H(C)	−102.95	−102.62
Cl_3_Pb···NH_3_CH_3_	Cl···H(N)	−115.95	−115.66
Cl_3_Pb···CH_3_NH_3_	Cl···H(C)	−82.78	−82.55
Cl_3_Pb···H_3_NCH_3_	Cl···H(N), Cl···H(C)	−107.24	−106.99
F_3_Pb···NH_3_CH_3_	F···H(N)	−138.54	−138.23
F_3_Pb···CH_3_NH_3_	F···H(C)	−94.39	−94.18
F_3_Pb···H_3_NCH_3_	F···H(N), F···H(C)	−126.47	−126.2
I_3_Sn···NH_3_CH_3_	I···H(N)	−103.12	−103.01
I_3_Sn···CH_3_NH_3_	I···H(C)	−75.02	−74.93
I_3_Sn···H_3_NCH_3_	I···H(N), I···H(C)	−96.07	−95.96
Br_3_Sn···NH_3_CH_3_	Br···H(N)	−108.39	−108.01
Br_3_Sn···CH_3_NH_3_	Br···H(C)	−78.17	−77.89
Br_3_Sn···H_3_NCH_3_	Br···H(N), Br···H(C)	−100.76	−100.44
Cl_3_Sn···NH_3_CH_3_	Cl···H(N)	−112.48	−112.19
Cl_3_Sn···CH_3_NH_3_	Cl···H(C)	−80.43	−80.2
Cl_3_Sn···H_3_NCH_3_	Cl···H(N), Cl···H(C)	−104.45	−104.19
F_3_Sn···NH_3_CH_3_	F···H(N)	−131.44	−131.11
F_3_Sn···CH_3_NH_3_	F···H(C)	−90.37	−90.15
F_3_Sn···H_3_NCH_3_	F···H(N), F···H(C)	−121.08	−120.79
I_3_Ge···NH_3_CH_3_	I···H(N)	−101.91	−101.74
I_3_Ge···CH_3_NH_3_	I···H(C)	−74.2	−74.05
I_3_Ge···H_3_NCH_3_	I···H(N), I···H(C)	−95.31	−95.13
Br_3_Ge···NH_3_CH_3_	Br···H(N)	−106.63	−106.22
Br_3_Ge···CH_3_NH_3_	Br···H(C)	−77.07	−76.74
Br_3_Ge···H_3_NCH_3_	Br···H(N), Br···H(C)	−99.64	−99.26
Cl_3_Ge···NH_3_CH_3_	Cl···H(N)	−110.15	−109.82
Cl_3_Ge···CH_3_NH_3_	Cl···H(C)	−79.01	−78.74
Cl_3_Ge···H_3_NCH_3_	Cl···H(N), Cl···H(C)	−102.88	−102.56
F_3_Ge···NH_3_CH_3_	F···H(N)	−125.21	−124.92
F_3_Ge···CH_3_NH_3_	F···H(C)	−87.26	−87.04
F_3_Ge···H_3_NCH_3_	F···H(N), F···H(C)	−116.94	−116.66
I_3_Si···NH_3_CH_3_	I···H(N)	−100.36	−100.25
I_3_Si···CH_3_NH_3_	I···H(C)	−73.01	−72.91
Br_3_Si···NH_3_CH_3_	Br···H(N)	−104.19	−103.86
Br_3_Si···CH_3_NH_3_	Br···H(C)	−75.28	−75.03
Cl_3_Si···NH_3_CH_3_	Cl···H(N)	−106.61	−106.34
Cl_3_Si···CH_3_NH_3_	Cl···H(C)	−76.58	−76.37
Cl_3_Si···H_3_NCH_3_	Cl···H(N), Cl···H(C)	−99.92	−99.65
F_3_Si···NH_3_CH_3_	F···H(N)	−115.3	−115.02
F_3_Si···CH_3_NH_3_	F···H(C)	−81.46	−81.24
F_3_Si···CH_3_NH_3_	F···H(N), F···H(C)	−109.02	−108.77

**Table 3:** Selected QTAIM-based topological charge density properties associated with the tetrel and pnictogen bonds of [CH_3_NH_3_^+^•TtX_3_^−^]_2_ (Tt = Pb, Sn, Ge, Si; X = I, Br, Cl, F), obtained with [*ω*B97X-D/def2-TZVPPD]. Values in a.u. ^a,b^.

System	Tetrel Bond	*ρ*_b_	∇^2^*ρ*_b_	*H*_b_	Pnictogen Bond	*ρ*_b_	∇^2^*ρ*_b_	*H*_b_
[CH_3_NH_3_^+^•PbI_3_^−^]_2_	Pb···I	0.0144	0.0272	0.0002	N···I	0.0058	0.0189	0.0009
[CH_3_NH_3_^+^•PbBr_3_^−^]_2_	Pb···Br	0.0163	0.0368	0.0003	N···Br	0.0064	0.0230	0.0011
[CH_3_NH_3_^+^•PbCl_3_^−^]_2_	Pb···Cl	0.0187	0.0496	0.0007	N···Cl	0.0066	0.0257	0.0013
[CH_3_NH_3_^+^•PbF_3_^−^]_2_	Pb···F	0.0342	0.1373	0.0002	N···F	0.0062	0.0294	0.0016
[CH_3_NH_3_^+^•SnI_3_^−^]_2_	Sn···I	0.0108	0.0191	0.0003	N···I	0.0070	0.0231	0.0010
[CH_3_NH_3_^+^•SnBr_3_^−^]_2_	Sn···Br	0.0113	0.0234	0.0004	N···Br	0.0080	0.0293	0.0012
[CH_3_NH_3_^+^•SnCl_3_^−^]_2_	Sn···Cl	0.0124	0.0292	0.0008	N···Cl	0.0086	0.0339	0.0015
[CH_3_NH_3_^+^•SnF_3_^−^]_2_	Sn···F	0.0283	0.0965	0.00005	N···F	0.0094	0.0463	0.0023
[CH_3_NH_3_^+^•GeI_3_^−^]_2_	Ge···I	0.0085	0.0193	0.0007	N···I	0.0077	0.0253	0.0010
[CH_3_NH_3_^+^•GeBr_3_^−^]_2_	Ge···Br	0.0090	0.0174	0.0005	N···Br	0.0090	0.0326	0.0013
[CH_3_NH_3_^+^•GeCl_3_^−^]_2_	Ge···Cl	0.0082	0.0211	0.0010	N···Cl	0.0096	0.0378	0.0016
[CH_3_NH_3_^+^•GeF_3_^−^]_2_	Ge···F	0.0125	0.0415	0.0014	N···F	0.0116	0.0594	0.0027
[CH_3_NH_3_^+^•SiI_3_^−^]_2_	Si···I	0.0068	0.0134	0.0005	N···I	0.0083	0.0274	0.0190
[CH_3_NH_3_^+^•SiBr_3_^−^]_2_	Si···Br	0.0054	0.0125	0.0006	N···Br	0.0096	0.0351	0.0263
[CH_3_NH_3_^+^•SiCl_3_^−^]_2_	Si···Cl	0.0026	0.0063	0.0004	N···Cl	0.0105	0.0418	0.0376
[CH_3_NH_3_^+^•SiF_3_^−^]_2_	H···Si ^b^	0.0228	0.0281	−0.0018	N···F	0.0102	0.0496	0.0669

^a^ The properties include the charge density (*ρ*_b_), the Laplacian of the charge density (∇^2^*ρ*_b_) and the total energy density (*H*_b_). ^b^ A hydrogen bond (see Figure S11d).

**Table 4:** [*ω*B97X-D/def2-TZVPPD] level uncorrected and BSSE-corrected interaction energies, Δ*E* and Δ*E*(BSSE), respectively (kcal mol^−1^), for the [CH_3_NH_3_^+^•TtX_3_^−^]_2_ (Tt = Pb, Sn, Ge, Si; X = I, Br, Cl, I) binary complexes examined.

System	Interaction Type ^a^	Δ*E*	Δ*E*(BSSE)
[CH_3_NH_3_^+^•PbI_3_^−^]_2_	Pb···I	−17.11	−16.83
[CH_3_NH_3_^+^•PbBr_3_^−^]_2_	Pb···Br	−17.78	−17.26
[CH_3_NH_3_^+^•PbCl_3_^−^]_2_	Pb···Cl	−18.26	−17.85
[CH_3_NH_3_^+^•PbF_3_^−^]_2_	Pb···F	−25.21	−24.73
[CH_3_NH_3_^+^•SnI_3_^−^]_2_	Sn···I	−13.80	−13.51
[CH_3_NH_3_^+^•SnBr_3_^−^]_2_	Sn···Br	−13.40	−12.87
[CH_3_NH_3_^+^•SnCl_3_^−^]_2_	Sn···Cl	−12.99	−12.59
[CH_3_NH_3_^+^•SnF_3_^−^]_2_	Sn···F	−17.22	−16.72
[CH_3_NH_3_^+^•GeI_3_^−^]_2_	Ge···I	−12.11	−11.66
[CH_3_NH_3_^+^•GeBr_3_^−^]_2_	Ge···Br	−11.08	−10.41
[CH_3_NH_3_^+^•GeCl_3_^−^]_2_	Ge···Cl	−10.01	−9.50
[CH_3_NH_3_^+^•GeF_3_^−^]_2_	Ge···F	−9.09	−8.60
[CH_3_NH_3_^+^•SiI_3_^−^]_2_	Si···I	−10.20	−9.92
[CH_3_NH_3_^+^•SiBr_3_^−^]_2_	Si···Br	−8.81	−8.33
[CH_3_NH_3_^+^•SiCl_3_^−^]_2_	Si···Cl	−7.49	−7.15
[CH_3_NH_3_^+^•SiF_3_^−^]_2_	H···Si ^b^	−14.48	−14.35

^a^ See text for discussion. ^b^ A hydrogen bond (see Figure S11d).

## Data Availability

This research did not report any data.

## Supplementary Materials

The following supporting information can be downloaded at: https://www.mdpi.com/article/10.3390/ijms241310554/s1.
