# Supplementary material for: Methylammonium Tetrel Halide Perovskite Ion Pairs and Their Dimers: The Interplay between the Hydrogen-, Pnictogen- and Tetrel-Bonding Interactions
Source: Int J Mol Sci. 2023 Jun 23;24(13):10554. doi: 10.3390/ijms241310554 (PMC10341982; doi:10.3390/ijms241310554)
Supplement: Supplementary file 1 [file ijms-24-10554-s001.zip › ijms-2412034-supplementary.pdf]

Methylammonium Tetrel Halide Perovskite Ion pairs and Their Dimers: The Interplay Between the Hydrogen-, Pnictogen- and Tetrel-Bonding Interactions

Pradeep R. Varadwaj[1,2,*] Arpita Varadwaj,[1*] Helder M. Marques[2], Koichi Yamashita[1]

[1] Department of Chemical System Engineering, School of Engineering, The University of Tokyo 7-3-1, Tokyo 113-8656, Japan
[2] Molecular Sciences Institute, School of Chemistry, University of the Witwatersrand, Johannesburg 2050, South Africa
* Correspondence: pradeep@t.okayama-u.ac.jp (PRV); varadwaj.arpita@gmail.com (A.V.)

Electronic Supplementary Information

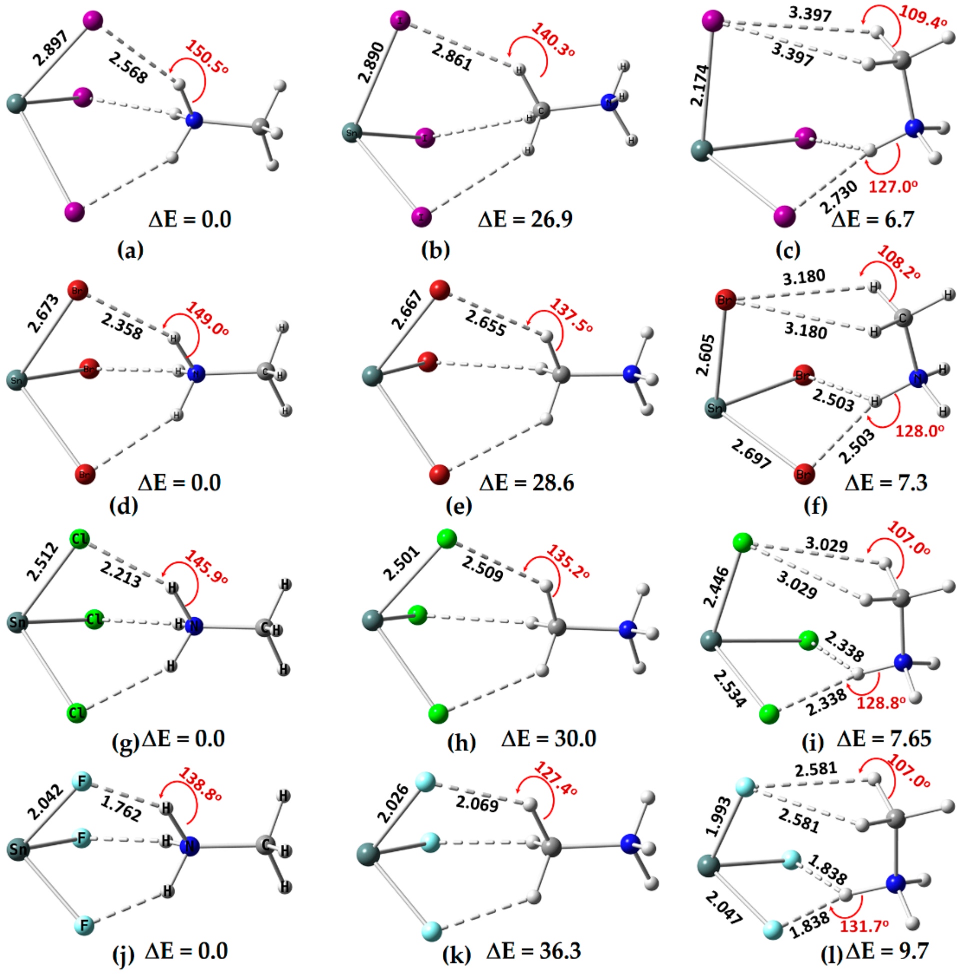

Figure S1. [*ω*B97XD/def2-TZVPPD] level fully-relaxed geometries of [$CH_3NH_3^+$•SnX_3^-] (X = F, Cl, Br, I) ion-pairs in the conformational space, including (a) [$I_3Sn\cdots NH_3CH_3$]; (b)

$[I_3Sn\cdots CH_3NH_3]$; (c) $[Br_3Sn\cdots NH_3CH_3]$; (d) $[Br_3Sn\cdots CH_3NH_3]$; (e) $[Cl_3Sn\cdots NH_3CH_3]$; (f) $[Cl_3Sn\cdots CH_3NH_3]$; (g) $[Cl_3Sn\cdots H_3NCH_3]$. Shown are selected coordinate and hydrogen bond distances in Å and hydrogen bond angles in degrees. The relative energies (ΔE) with respect to the most stable conformer is shown in kcal mol^{-1}. Atom labeling is shown for selected systems.

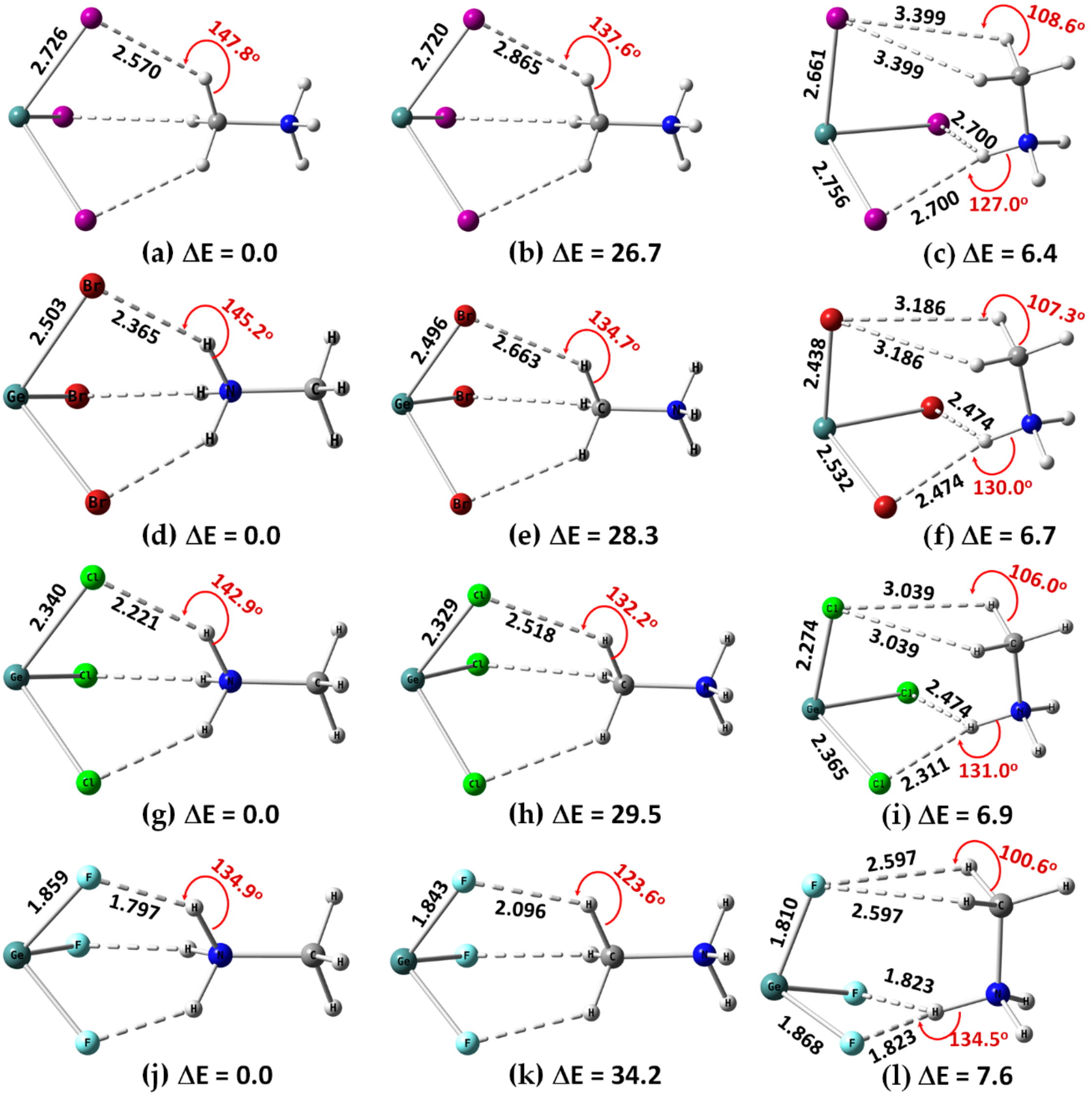

Figure S2. [*ω*B97XD/def2-TZVPPD] level fully-relaxed geometries of [$CH_3NH_3^+$•GeX_3^-] (X = F, Cl, Br, I) ion-pairs in the conformational space, including (a) [$I_3Ge\cdots NH_3CH_3$]; (b) [$I_3Ge\cdots CH_3NH_3$]; (c) [$Br_3Ge\cdots NH_3CH_3$]; (d) [$Br_3Ge\cdots CH_3NH_3$]; (e) [$Cl_3Ge\cdots NH_3CH_3$]; (f) [$Cl_3Ge\cdots CH_3NH_3$]; (g) [$Cl_3Ge\cdots H_3NCH_3$]. Shown are selected coordinate and hydrogen bond distances in Å and hydrogen bond angles in degrees. The relative energies (Δ*E*) with respect to the most stable conformer is shown in kcal mol^{-1}. Atom labeling is shown for selected systems

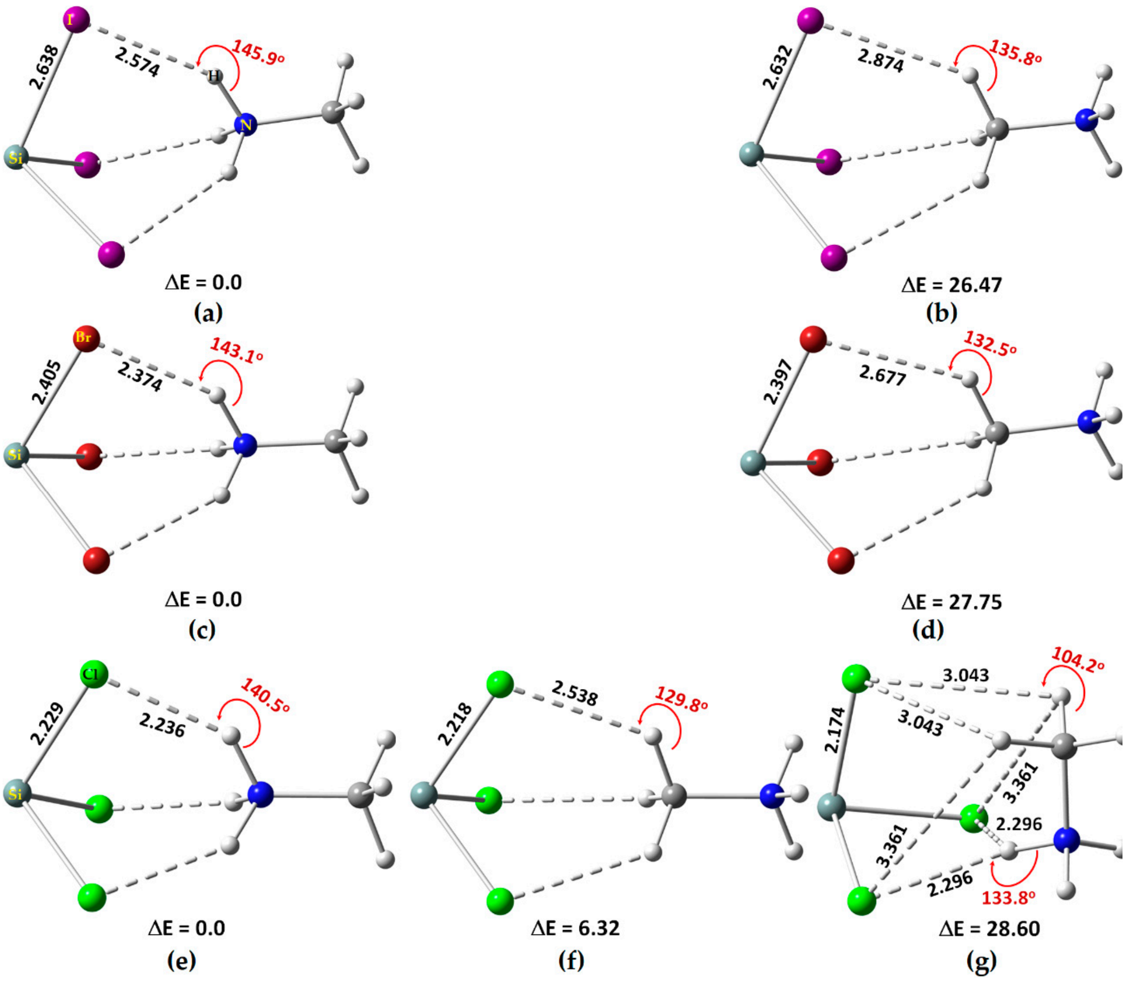

Figure S3. [ωB97XD/def2-TZVPPD] level fully-relaxed geometries of [$CH_3NH_3^+ \bullet SiX_3^-$] ($X$ = Cl, Br, I) ion-pairs in the conformational space, including (a) [$I_3Si \cdots NH_3CH_3$]; (b) [$I_3Si \cdots CH_3NH_3$]; (c) [$Br_3Si \cdots NH_3CH_3$]; (d) [$Br_3Si \cdots CH_3NH_3$]; (e) [$Cl_3Si \cdots NH_3CH_3$]; (f) [$Cl_3Si \cdots CH_3NH_3$]; (g) [$Cl_3Si \cdots H_3NCH_3$]. Shown are selected coordinate and hydrogen bond distances in Å and hydrogen bond angles in degrees. The relative energies (ΔE) with respect to the most stable conformer is shown in kcal mol^{-1}. Atom labeling is shown for selected systems.

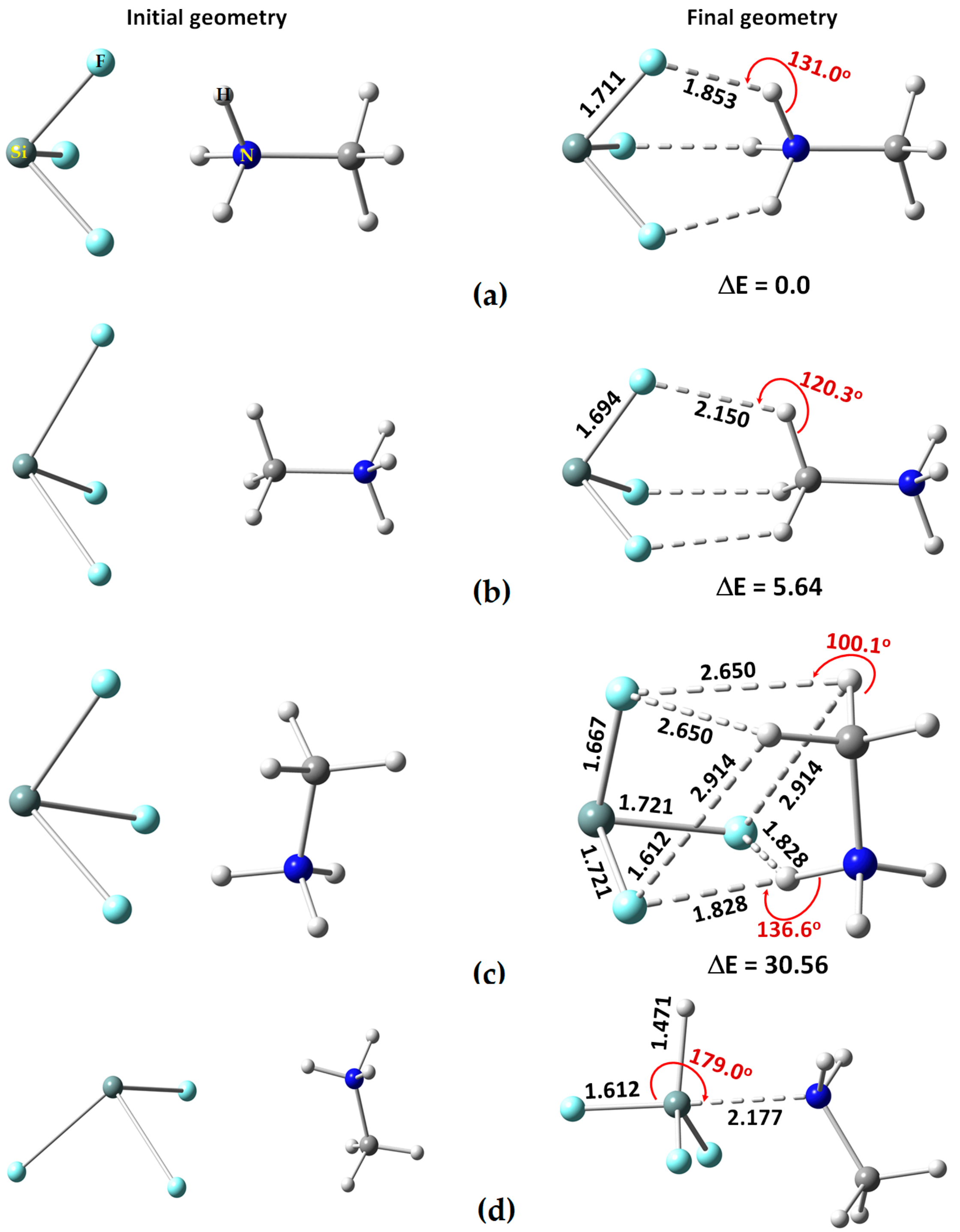

Figure S4. Initial (left) and final [ωB97XD/def2-TZVPPD] (right) configurations of [$CH_3NH_3^+$•SiF_3^-]. These include: (a) [$F_3Si\cdots NH_3CH_3$]; (b) [$F_3Si\cdots CH_3NH_3$]; (c) [$F_3Si\cdots H_3NCH_3$]; (d) $F_3HSi\cdots NH_2CH_3$. Shown (a)-(c) (right) are selected coordinate and hydrogen bond distances in Å and hydrogen bond angles in degrees. The relative energies (ΔE) with respect to the most stable conformer (a) is shown in kcal mol^{-1}. Atom labeling is shown in (a). Shown in (d) are selected coordinate bond and tetrel bond distances in Å and tetrel bond angle in degrees.

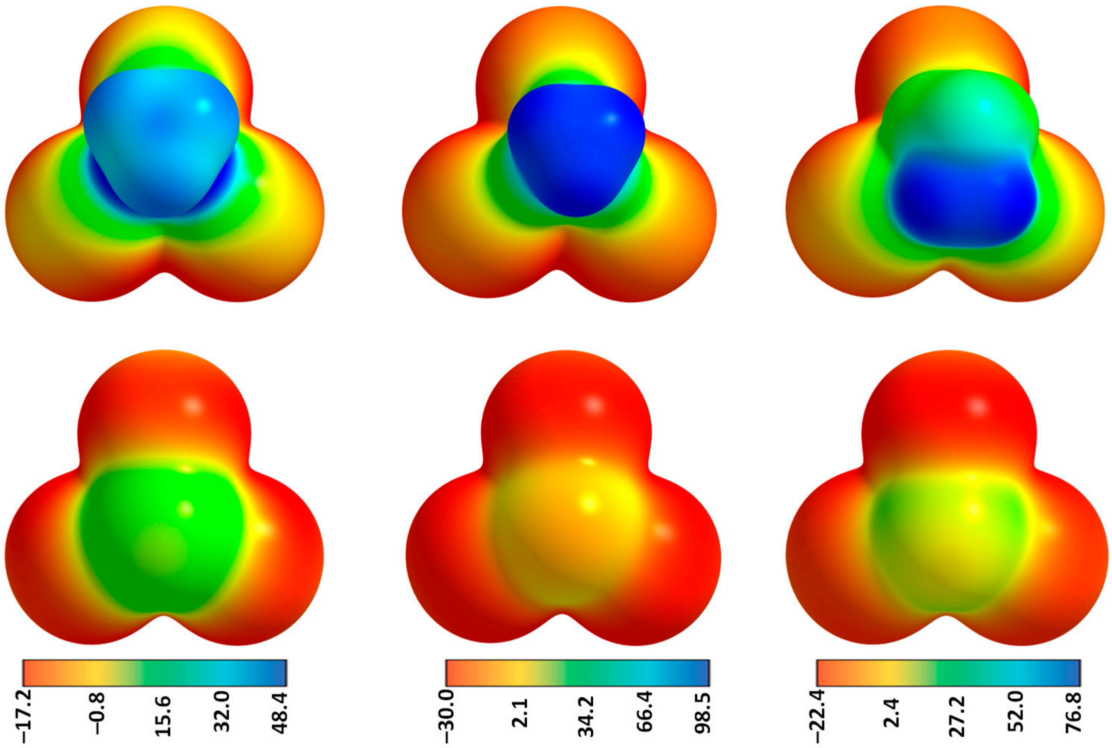

$[SnI_3^{-}\cdots NH_3CH_3^{+}]$ **(a) Conf 1** $[SnI_3^{-}\cdots CH_3NH_3^{+}]$ **(c) Conf 2** $[SnI_3^{-}\cdots H_3NCH_3^{+}]$ **(b) Conf 3**

Figure S5. [*ω*B97XD/def2-TZVPPD] level potential on the electrostatic surface of the three conformations of $[CH_3NH_3^{+}\bullet SnI_3^{-}]$: (a) $[SnI_3^{-}\cdots NH_3CH_3^{+}]$; (b) $[SnI_3^{-}\cdots CH_3NH_3^{+}]$; and (c) $[SnI_3^{-}\cdots H_3NCH_3^{+}]$. The methyl, ammonium and both groups of MA are facing the reader in the top panel of (a), (b) and (c), respectively. The Sn atom is facing the reader in the bottom panel of all the three MESP plots shown in (a)-(c). Values on the color bar are in kcal mol^{-1}. Atom labeling is shown for selected systems

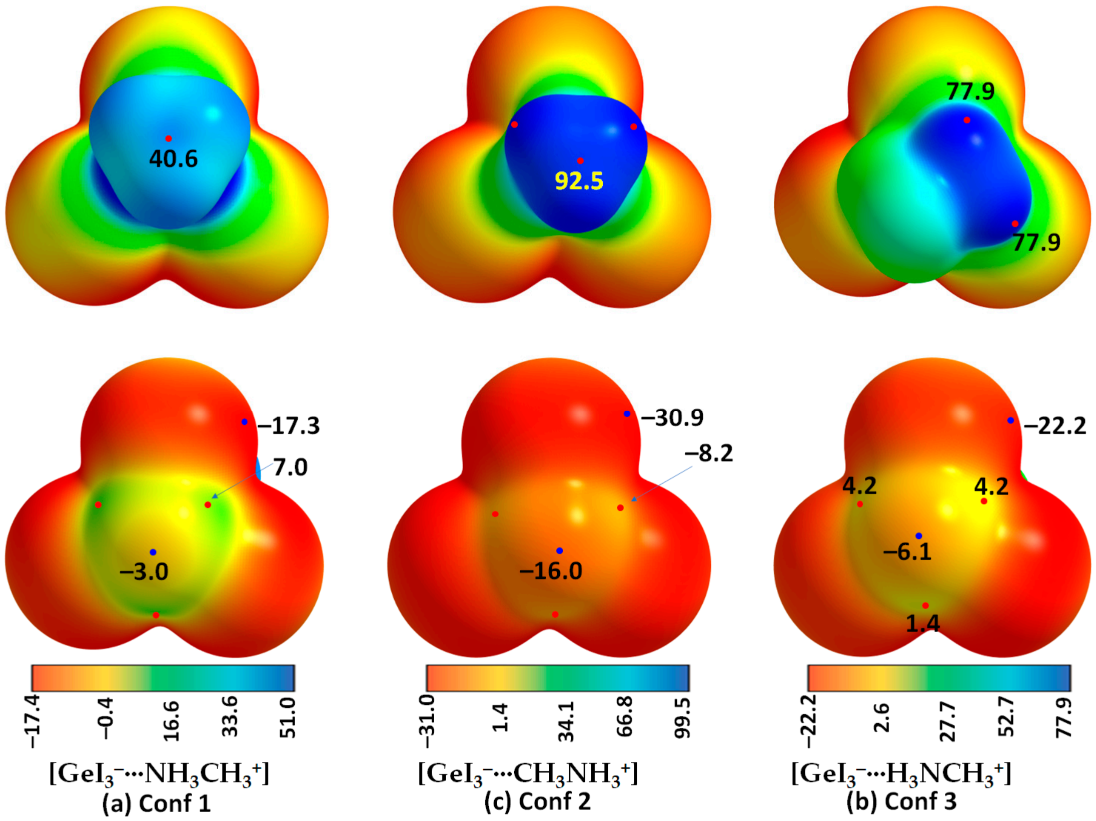

Figure S6. [ωB97XD/def2-TZVPPD] level potential on the electrostatic surface of the three conformations of [$CH_3NH_3^+$•GeI_3^-]: (a) [GeI_3^-···$NH_3CH_3^+$]; (b) [GeI_3^-···$CH_3NH_3^+$]; and (c) [GeI_3^-···$H_3NCH_3^+$]. The methyl, ammonium and both groups of MA are facing the reader in the top panel of (a), (b) and (c), respectively. The Sn atom is facing the reader in the bottom panel of all the three MESP plots shown in (a)-(c). Values on the color bar are in kcal mol^{-1}. Atom labeling is shown for selected systems.

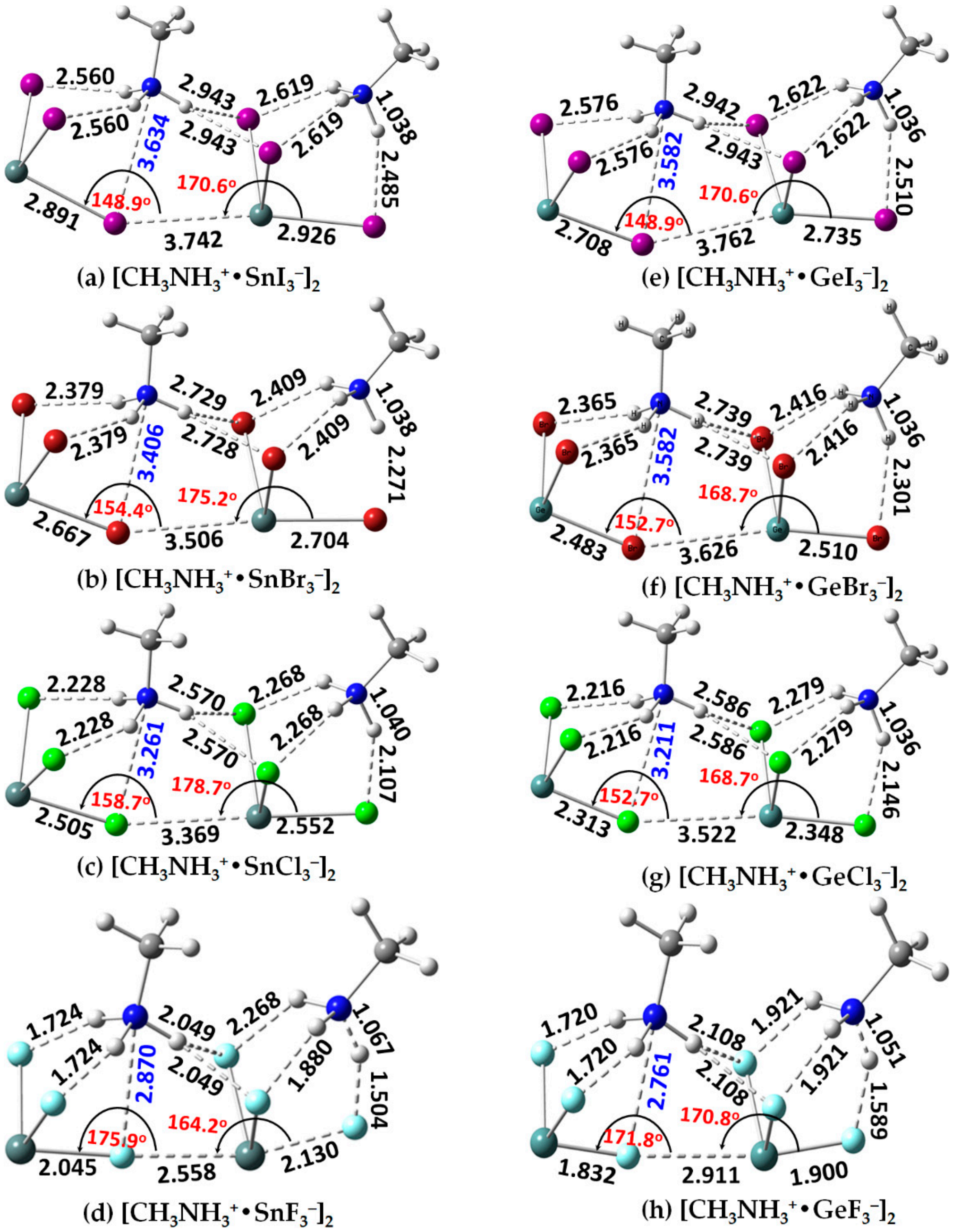

Figure S7. [ωB97XD/def2-TZVPPD] level fully relaxed gas-phase geometries of (a) $[CH_3NH_3^+ \bullet SnI_3^-]_2$; (b) $[CH_3NH_3^+ \bullet SnBr_3^-]_2$; (c) $[CH_3NH_3^+ \bullet SnCl_3^-]_2$; and (d) $[CH_3NH_3^+ \bullet SnF_3^-]_2$; (e) $[CH_3NH_3^+ \bullet GeI_3^-]_2$; (f) $[CH_3NH_3^+ \bullet GeBr_3^-]_2$; (g) $[CH_3NH_3^+ \bullet GeCl_3^-]_2$; and (d) $[CH_3NH_3^+ \bullet GeF_3^-]_2$. Selected bond lengths (Å) and bond angles (degrees) are shown in (a)-(h).

.

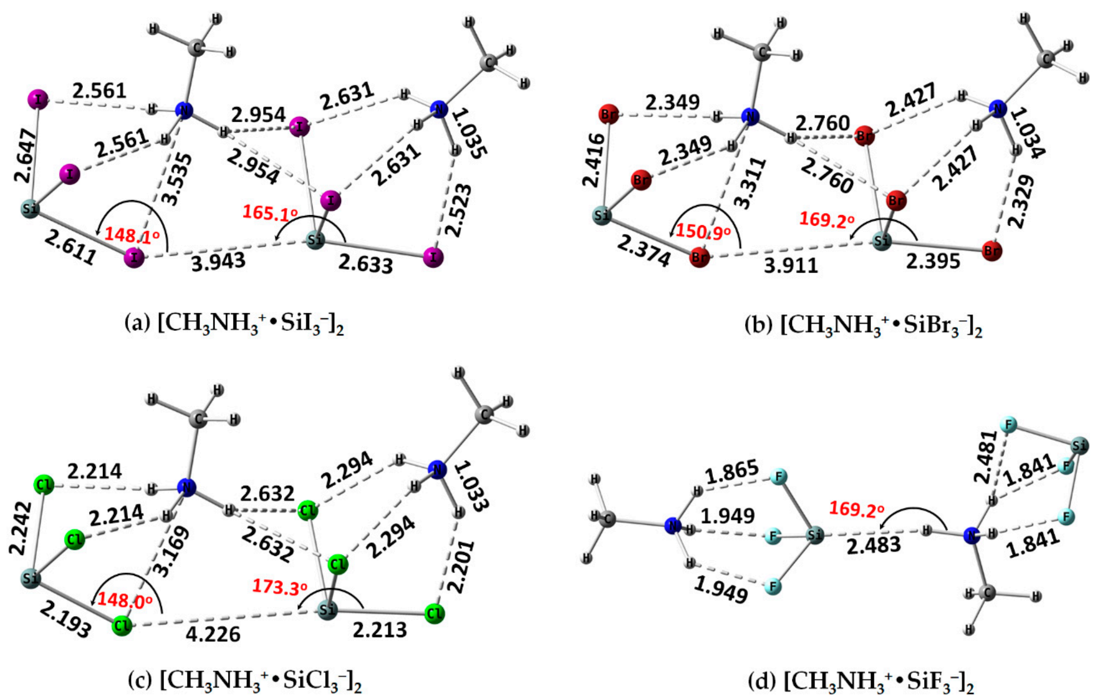

Figure S8. [ωB97XD/def2-TZVPPD] level fully relaxed gas-phase geometries of (a) $[CH_3NH_3^+ \bullet SiI_3^-]_2$; (b) $[CH_3NH_3^+ \bullet SiBr_3^-]_2$; (c) $[CH_3NH_3^+ \bullet SiCl_3^-]_2$; and (d) $[CH_3NH_3^+ \bullet SiF_3^-]_2$. Selected bond lengths (Å) and bond angles (degrees) are shown in (a)-(h). Atom labeling is shown for each case.

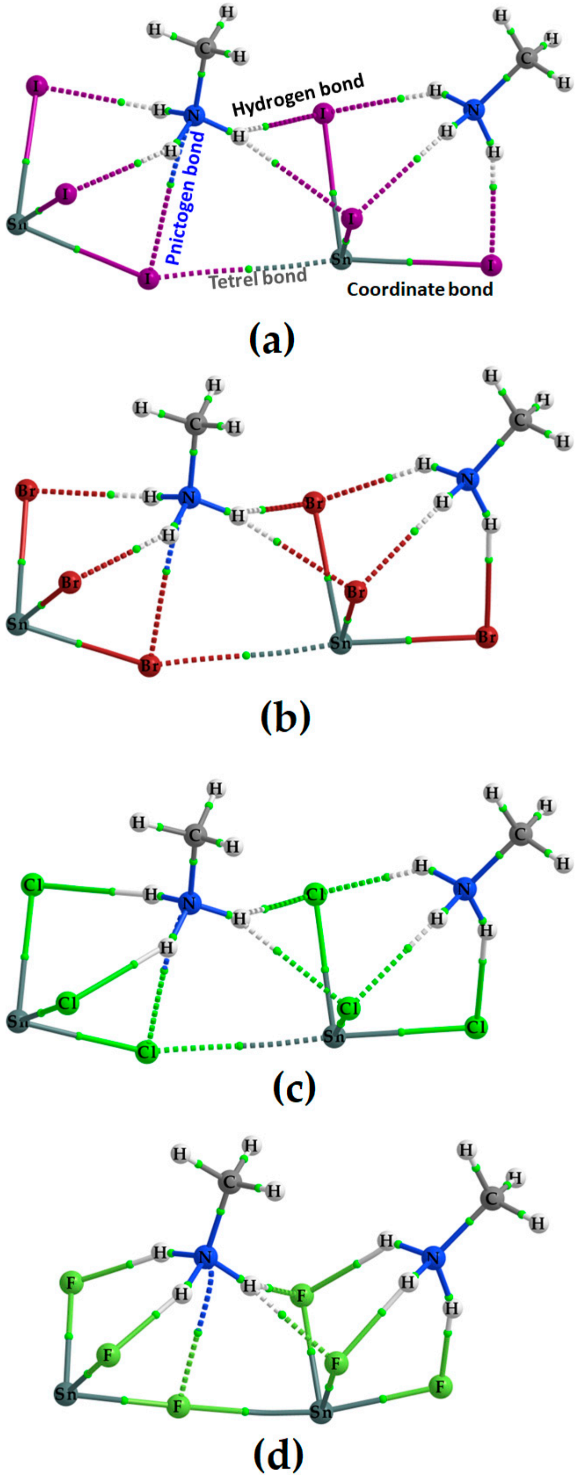

Figure S9. [ωB97XD/def2-TZVPPD] computed QTAIM based molecular graphs of a) $[CH_3NH_3^+\bullet SnI_3^-]_2$; (b) $[CH_3NH_3^+\bullet SnBr_3^-]_2$; (c) $[CH_3NH_3^+\bullet SnCl_3^-]_2$; and (d) $[CH_3NH_3^+\bullet SnF_3^-]_2$. The bond critical points between atomic basins are shown as tiny spheres in green, and bond paths as solid and lines in atom color. The values (in a.u.) of the charge density (ρ_b), the Laplacian of the charge density ($\nabla^2\rho_b$), and the total energy density (H_b) are given for selected bcps. Bond type and atom labeling is shown

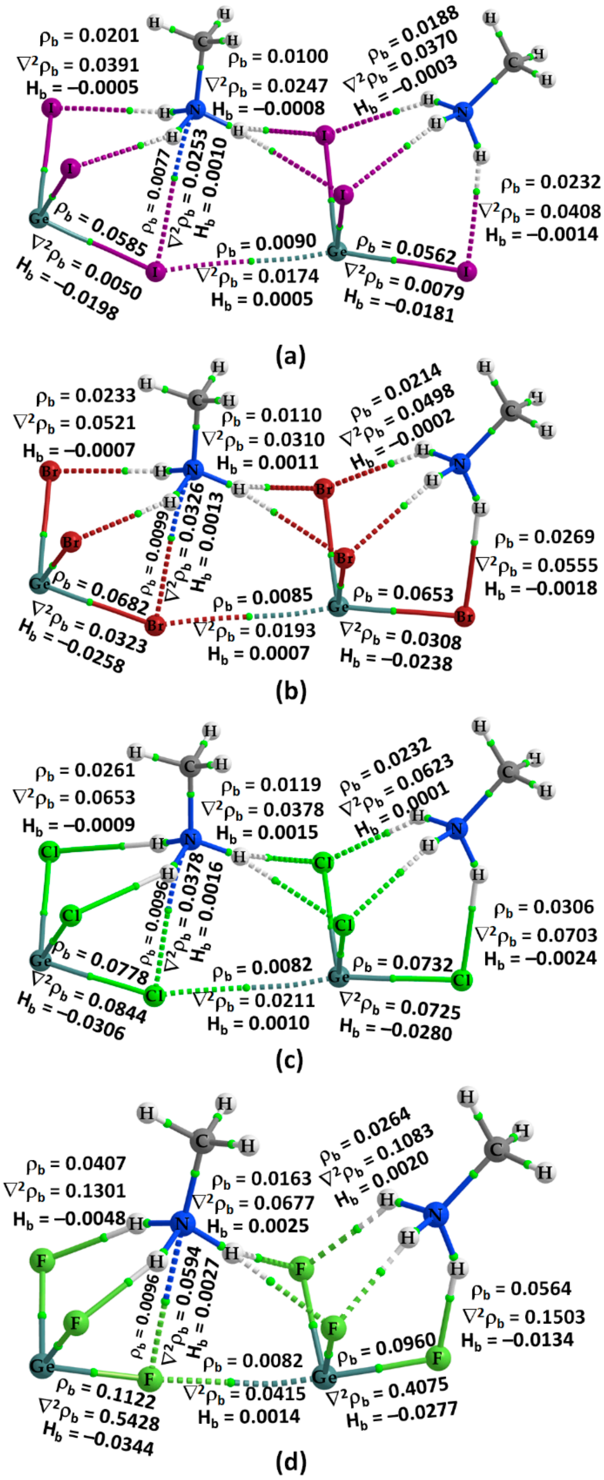

Figure S10. [ωB97XD/def2-TZVPPD] computed QTAIM based molecular graphs of a) $[CH_3NH_3^+\bullet GeI_3^-]_2$; (b) $[CH_3NH_3^+\bullet GeBr_3^-]_2$; (c) $[CH_3NH_3^+\bullet GeCl_3^-]_2$; and (d) $[CH_3NH_3^+\bullet GeF_3^-]_2$. The bond critical points between atomic basins are shown as tiny spheres in green, and bond paths as solid and lines in atom color. The values (in a.u.) of the charge density (ρ_b), the Laplacian of the charge density ($\nabla^2\rho_b$), and the total energy density (H_b) are given for selected bcps. Bond type and atom labeling is shown.

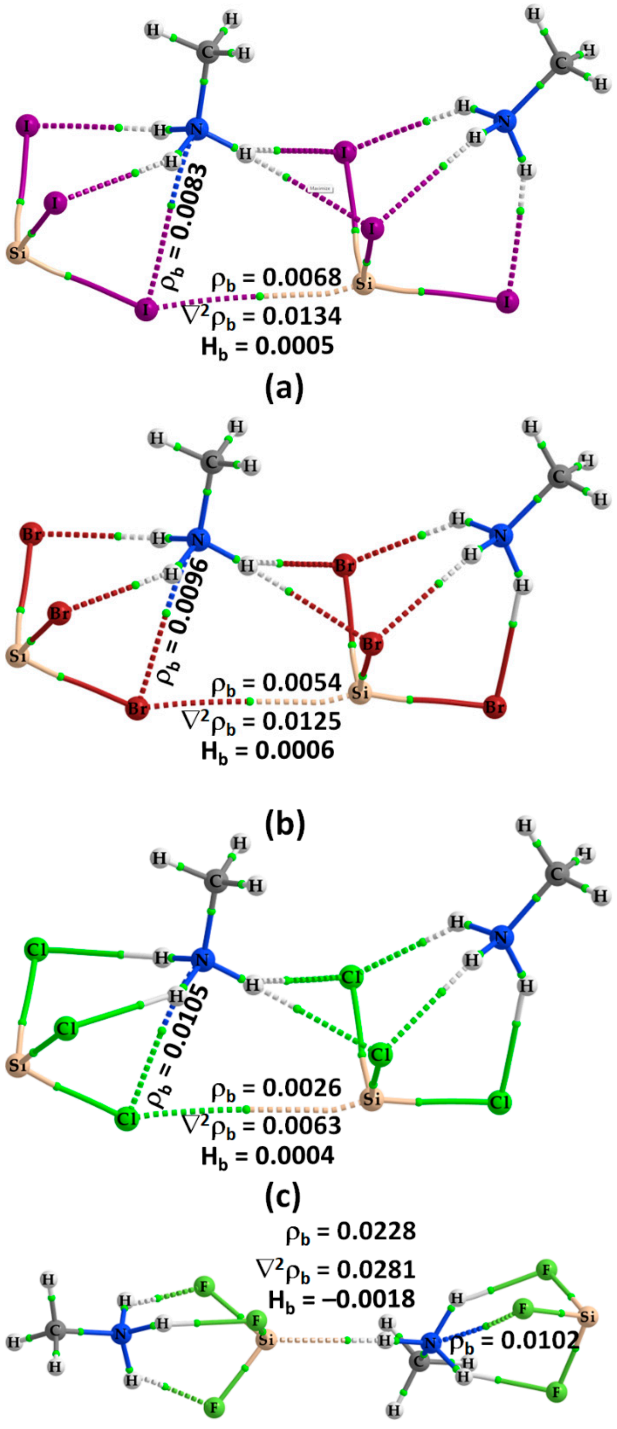

Figure S11. [ωB97XD/def2-TZVPPD] computed QTAIM based molecular graphs of a) $[CH_3NH_3^+ \bullet SiI_3^-]_2$; (b) $[CH_3NH_3^+ \bullet SiBr_3^-]_2$; (c) $[CH_3NH_3^+ \bullet SiCl_3^-]_2$; and (d) $[CH_3NH_3^+ \bullet SiF_3^-]_2$. The bond critical points between atomic basins are shown as tiny spheres in green, and bond paths as solid and lines in atom color. Bond type and atom labeling is shown. Charge density properties (ρ_b, $\nabla^2\rho_b$, and H_b) in a.u. are shown for selected bcps.

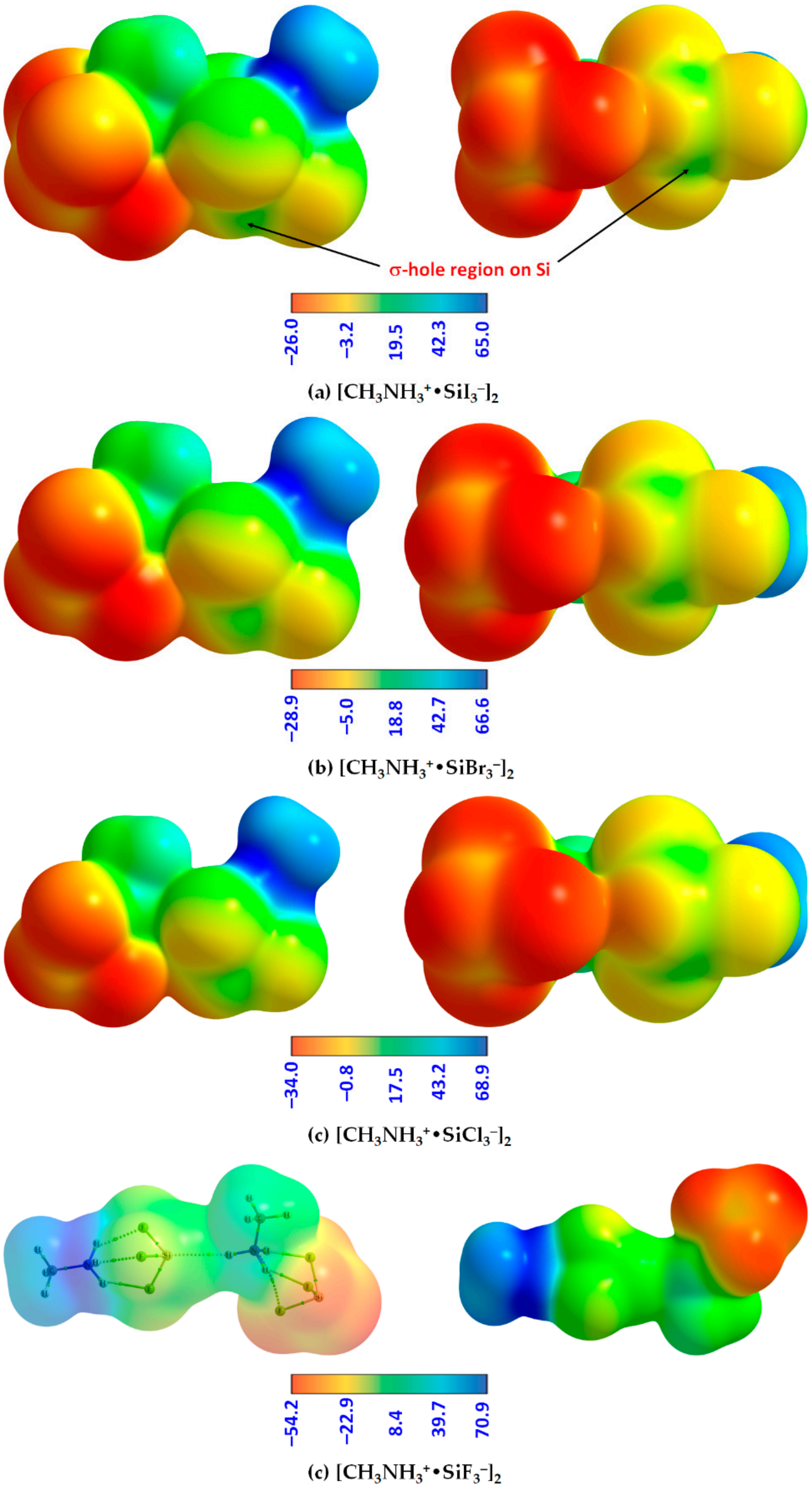

Figure S12. Two different views (left and right) of [ωB97XD/def2-TZVPPD] computed 0.001 a.u. mapped electrostatic potential on the molecular surfaces of a) $[CH_3NH_3^+ \bullet SiI_3^-]_2$; (b) $[CH_3NH_3^+ \bullet SiBr_3^-]_2$; (c) $[CH_3NH_3^+ \bullet SiCl_3^-]_2$; and (d) $[CH_3NH_3^+ \bullet SiF_3^-]_2$. Values on the color bar are in kcal mol^{-1}.

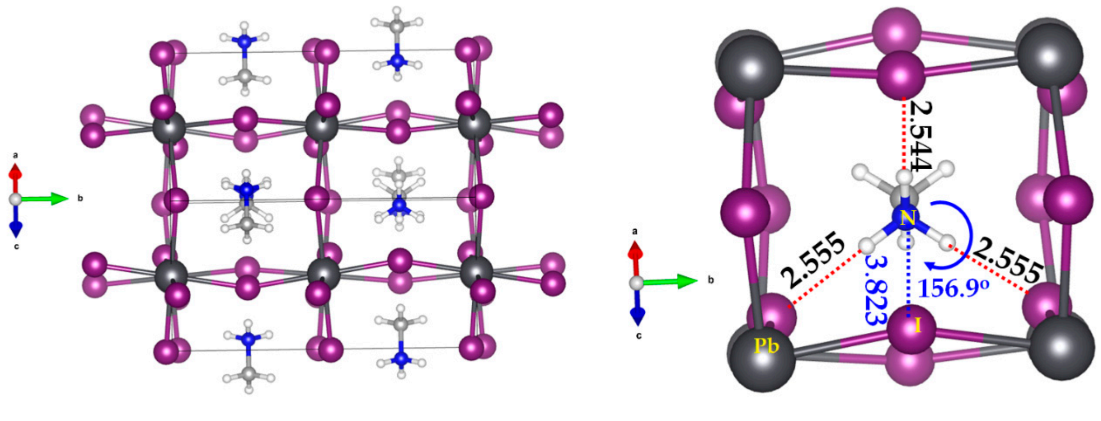

(a) o-MAPbI$_3$

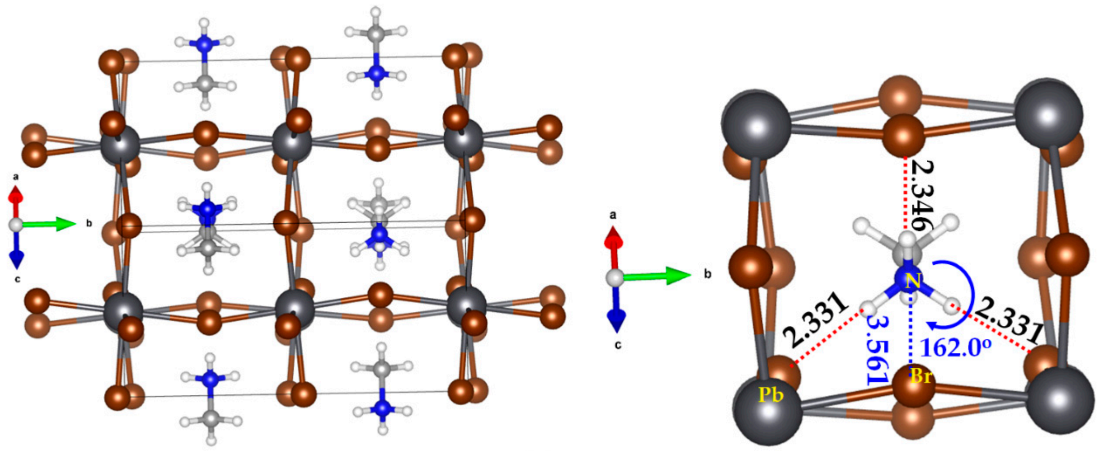

(b) o-MAPbBr$_3$

Figure S13. (a) (left) [PBEsol/PAW] relaxed unit-cell geometry of MAPbI$_3$, showing (right) the nature of N–H···I hydrogen bond and C–N···I pnictogen bond. The pnictogen bond in the crystal (ICSD ref: 428898) of the corresponding has bond distance and bond angle of 3.786 Å and 152.6^{o}, respectively, whereas the three N–H···I hydrogen bonds were 2.807, 2.807 and 2.613 Å. Shown in (b) is the [PBEsol/PAW] relaxed unit-cell geometry of MAPbBr$_3$, showing (right) the nature of N–H···Br hydrogen bond and C–N···Br pnictogen bond; the bond distance and bond angle of the latter contact in the crystal (ICSD ref: 268782), which has the H atoms in MA missing, were 3.570 Å and 155.0^{o}, respectively. A k-mesh of 4 × 4 × 4, together with the global break condition for the electronic self-consistent and ionic relaxation loops were 10^{-6} and -10^{-2} eV, respectively, was used. The VASP (version 5.4) code was used.

Table S1. Selected [MP2/def2-TZVPPD] computed 0.001 a.u. isoelectronic density envelope mapped maxima and minima of potential (kcal mol^{-1}) on the electrostatic surfaces of X_3Pb^{-}···MA ion-pairs, where X = I, Br, Cl, F, and MA = $NH_3CH_3^{+}$.[a]

Ion-pairs	$V_{S,max}$(X–Pb)	$V_{S,max}$(X–Pb)	$V_{S,max}$(X–Pb)	$V_{S,min}$(Pb)	$V_{S,max}$(N-C)/$V_{S,max}$(C-N)	$V_{S,min}$ (X)
I_3Pb···NH_3CH_3	25.7	25.7	25.7	23.8	36.2	-17.4
Br_3Pb···CH_3NH_3	27.3	27.3	27.3	25.0	35.0	-20.3
Cl_3Pb···H_3NCH_3	26.9	26.9	26.9	24.3	33.7	-23.2
F_3Pb···NH_3CH_3	24.4	24.4	24.4	19.6	27.0	-37.1

[a] The repetition of $V_{S,max}$(X–Pb) three times suggest that they belong to three equivalent σ-holes along the outer extensions of three Pb–X bonds of the inorganic anion of an ion-pair.

Table S2. Selected [MP2/aug-cc-pVTZ] computed 0.001 a.u. isoelectronic density envelope mapped maxima and minima of potential (kcal mol^{-1}) on the electrostatic surfaces of X_3Pb^{-}···MA ion-pairs, where X = I, Br, Cl, F, and MA = $NH_3CH_3^{+}$.[a,b]

Ion-pairs	$V_{S,max}$(X–Pb)	$V_{S,max}$(X–Pb)	$V_{S,max}$(X–Pb)	$V_{S,min}$(Pb)	$V_{S,max}$(N-C)/$V_{S,max}$(C-N)	$V_{S,min}$ (X)
I_3Pb···NH_3CH_3	23.4	23.4	23.4	19.6	36.0	-16.0
Br_3Pb···CH_3NH_3	24.5	24.5	24.5	19.7	34.9	-19.1
Cl_3Pb···H_3NCH_3	24.3	24.3	24.3	18.8	34.4	-22.4
F_3Pb···NH_3CH_3	22.3	22.3	22.3	14.8	27.9	-36.0

[a] The pseudo-potential aug-cc-pVTZ-PP was used for Pb and I.

[b] The repetition of $V_{S,max}$(X–Pb) three times suggest that they belong to three equivalent σ-holes along the outer extensions of three Pb–X bonds of the inorganic anion of an ion-pair.
